# Loop-Mediated Isothermal Amplification for Influenza A (H5N1) Virus

**DOI:** 10.3201/eid1306.061572

**Published:** 2007-06

**Authors:** Shanthi Jayawardena, Chung Y. Cheung, Ian Barr, Kwok H. Chan, Honglin Chen, Yi Guan, J.S. Malik Peiris, Leo L.M. Poon

**Affiliations:** * The University of Hong Kong, Hong Kong Special Administrative Region, People’s Republic of China; † World Health Organization Collaborating Centre for Influenza, Melbourne, Victoria, Australia

**Keywords:** H5N1, avian influenza virus, molecular detection, loop-mediated isothermal amplification, dispatch

## Abstract

We describe a 1-step reverse-transcription loop-mediated isothermal amplification assay for detection of highly pathogenic avian influenza A (H5N1) viruses. The assay was tested by using a panel of highly pathogenic H5N1 subtypes isolated over the past 10 years and clinical specimens. The assay produced negative results for all non-H5N1 subtypes.

Highly pathogenic avian influenza A (H5N1) virus has had a significant global effect on the poultry industry, human healthcare, and many other sectors (*1*). Several molecular tests have been developed for the rapid detection of influenza (H5) virus subtypes (*2*), but they often require sophisticated equipment (e.g., PCR machine) and are difficult for researchers and clinicians to perform in resource-limited settings. Loop-mediated isothermal amplification (LAMP) provides a molecular testing option for this scenario (*3*). The LAMP mechanism has been described (*3*,*4*). Using this approach, nucleic acids are amplified under isothermal conditions (e.g., in a water bath) with high specificity, efficiency, and speed (*3*). The assay is highly specific due to recognition of target DNA by 6 independent sequences. An attractive feature of LAMP is its ability to generate large amounts of white magnesium pyrophosphate precipitate in positive reactions (*4*). Examples of positive and negative reactions are shown in Appendix Figure 1. LAMP enables easy visual identification of positive reactions (*4*,*5*) and avoids additional cost and labor for postamplification analysis. This closed-tube method can also minimize the problem of carryover contamination in less controlled environments.

## The Study

We aimed to develop a reverse transcription (RT) LAMP assay that could detect a variety of highly pathogenic influenza (H5N1) viruses. For primer design, we used highly pathogenic influenza (H5N1) sequences publicly available from the Influenza Sequence Database (www.flu.lanl.gov/index.html) in August 2006. We used hemagglutinin (HA) from an influenza (H5N1) prototype vaccine strain (A/Vietnam/1203/2004, GenBank accession no. AY651334) as the reference for this study. The sequences included highly pathogenic influenza (H5N1) viruses identified in the past 2 years. In addition, sequences from all the other HA subtypes (e.g., 1,127 for H1 and 1,472 for H3) in the above database were used for the analysis. Because highly pathogenic influenza (H5N1) gene sequences are genetically diverse, all studied H5 sequences (n = 711) were aligned, and a highly conserved region at the 5′ end of HA2 encoding sequence (corresponding to nt 1,065–1,298 of the reference sequence) was selected as the target (Appendix Figure2A, and Appendix Figure 3). Of all the downloaded highly pathogenic influenza (H5N1) sequences, 115 did not contain the target site and were excluded from analysis. The GenBank accession numbers for the highly pathogenic influenza (H5N1) sequences used for the primer design are listed in the Appendix Table.

Previous demonstration that degenerate primers could be used in LAMP assays (*6*) led us to use the same approach for designing a set of degenerate primers for the targeted region (Appendix Figure 2B). Our HA sequence analysis also showed that HA sequences from influenza (H5N2) viruses of North American lineage and other viruses with other HA subtypes contain extensive mismatches to the primers used in the LAMP assay (Appendix Figure 2A, B). In the initial phase of the study, we used the reference strain (A/Vietnam/1203/2004) to determine the sensitivity of the assay. Purified RNA from a viral supernatant with a known titer was serially diluted and subjected to the LAMP assay. As shown in the Figure panel A, the detection limit of the assay was found to be 2×10^–3^ plaque-forming units (pfu) per reaction. The sensitivity of this assay was also compared with that of an optimized RT-PCR assay recommended by the World Health Organization (*7*). As shown in the Figure panel A, the detection limit of the RT-PCR assay was identical to that from the LAMP assay. We also spiked various amounts of influenza (H5) viruses into non-H5 nasopharyngeal aspirate samples and tested the purified RNA from these clinical specimens by the LAMP and RT-PCR assays. The results from these assays were identical (data not shown). These findings are in accordance with previous findings that the sensitivity of LAMP assays is comparable to that of conventional PCR methods (*5*,*6*,*8*). In addition, all positive reactions were visually examined for the presence of white precipitate at the end of the reaction incubation. As expected, all results correlated exactly with those deduced by the real-time turbidity meter (data not shown).

**Figure:**
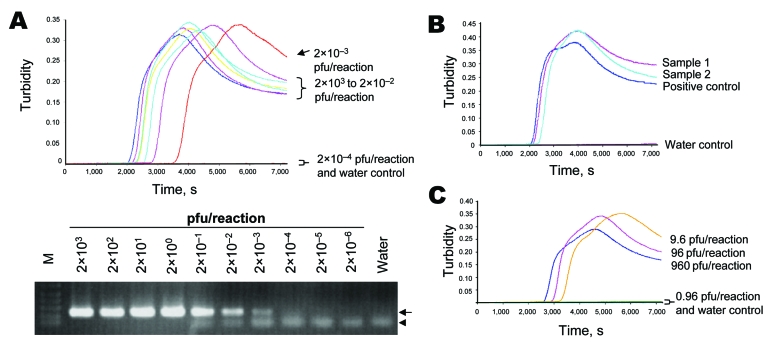
Detection of influenza (H5) virus by loop-mediated isothermal amplification (LAMP). A) Serially diluted RNA from A/Vietnam/1203/2004 was tested by the reverse transcription (RT)–LAMP (upper panel) and RT-PCR (lower panel) assays. The viral titers used in these reactions are indicated. Viral RNA was extracted by using the QIAamp Viral RNA Mini Kit (QIAGEN, Valencia, CA, USA) according to the manufacturer’s instructions. For a typical 25-μL reaction, 2 μL of sample was mixed with 2× in-house reaction buffer (40 mmol/L Tris-HCl, pH 8.8; 20 mmol/L KCl; 16 mmol/L MgSO_4_; 20 mmol/L [NH_4_]_2_SO_4_; 0.2% Tween 20 [v/v]; 1.6 mol/L betaine; 2.8 mmol/L each dNTP), 50 U *Bst* DNA polymerase (New England Biolabs, Ipswitch, MA, USA), 8 U avian myeloblastosis virus reverse transcriptase (Invitrogen, Gaithersburg, MD, USA), 40 pmol/L primers FIP and BIP, 20 pmol/L primers LPF and LPR, and 5 pmol/L primers F3 and B3. Reaction mixtures were incubated at 60ºC for 120 min, and the turbidity of these reactions was examined by use of a turbidity meter (LA-200, Treamecs; Kyoto, Japan) in real time. The turbidities of these reactions 5–20 min after incubation were taken as the baseline. The threshold value for a positive reaction was set to be 10× above the standard deviation of the baseline. For the H5-specific RT-PCR assay, primers H5-1 (5′-GCCATTCCACAACATACACCC-3′) and H5-3 (5′-CTCCCCTGCTCATTGCTATG-3′) were used according to the protocol optimized by the World Health Organization H5 Reference Laboratory Network (*7*). Positive (219 bp) and nonspecific products from the PCR reaction are highlighted by the arrow and arrowhead, respectively. B) Detection of H5 virus in postmortem lung tissues from a patient with influenza (H5). Signals from the tested samples, positive control, and water control are indicated. C) Direct detection of influenza (H5) viruses from culture supernatants. Heat-treated supernatant from cells infected with A/Vietnam/1203/2004 were serially diluted and directly used as input in the LAMP assay. The plaque-forming units (pfu) of influenza (H5) virus in these reactions are shown.

We also tested the feasibility of using this assay to detect influenza (H5) viruses in clinical specimens from a patient with influenza (H5). RNA from 2 different postmortem lung tissue samples from a patient infected with influenza A/HK/212/03 was subjected to the LAMP assay. As shown in the Figure panel B, both RNA samples had positive results for the influenza (H5) viruses.

To evaluate the specificity of the LAMP assay for influenza (H5) viruses, a comparative study including all 16 HA subtypes and a variety of H5N1 strains was performed (Table). All 14 H5N1 strains isolated from different geographic regions during the past 10 years were positive, whereas none of the non-H5 samples were positive (Table). All the reactions were visually examined, and the results corresponded with those generated from the real-time turbidity meter. Influenza A/chicken/Wajo/BBVM/2005, A/duck/Vietnam/568/2005, and A/bar-headed goose/Qinghai/5/2005 are clade 2 influenza (H5N1) viruses that have recently emerged from different geographic regions (*9*), and these 3 viruses are phylogenetically distinct (*10*,*11*). Our results demonstrate that this assay is applicable to a wide variety of highly pathogenic influenza (H5N1) viruses. In addition, other virus reassortants with the HA of this lineage are expected to be positive in the assay.

**Table:** RT-LAMP assay results for highly pathogenic  influenza A (H5N1)*

Virus subtype	Strain	Result
H1	A/HK/54/98	–
H2	A/Singapore/57	–
H3	A/HK/1174/99	–
H4	A/duck/HK/MPA892/06	–
H5N1	A/HK/483/97	+
H5N1	A/HK/486/97	+
H5N1	A/chicken/HK/61.9/2002	+
H5N1	A/goose/HK/739.2/2002	+
H5N1	A/HK/213/03	+
H5N1	A/HK/212/03	+
H5N1	A/Thailand/MK2/04	+
H5N1	A/Vietnam/1203/04	+
H5N1	A/chicken/Indoneasia/4/2004	+
H5N1	A/chicken/Thailand/1/2004	+
H5N1	A/chicken/Vietnam/33/2004	+
H5N1	A/chicken/Wajo/BBVM/2005	+
H5N1	A/duck/Vietnam/568/2005	+
H5N1	A/bar-headed goose/Qinghai/5/2005	+
H6	A/teal/HK/W312/97	–
H7	A/env/HK/MPB127/05	–
H8	A/duck/HK/MP4275/2005	–
H9	A/duck/HK/G1/97	–
H10	A/env/HK/MPB839/05	–
H11	A/env/HK/MPB1679/06	–
H12	A/red necked stint/WA/5745/1984	–
H13	A/gull/Maryland/704/1977	–
H14	A/mallard/Gurjev/244/1982	–
H15	A/shelduck/WA/1762/1979	–
H16	A/gull/Denmark/68110/2002	–

*RT-LAMP, reverse transcription loop-mediated isothermal amplification; –, negative; +, positive.

We previously demonstrated that heat-treated blood samples could be directly tested by a LAMP assay specific for DNA of a bloodborne pathogen (*5*). These modifications could avoid the need for nucleic acid purification, thereby reducing the cost and turnaround time for molecular diagnosis. We tested the feasibility of detecting the H5 sequence from viral cultures without RNA extraction. In a Biosafety Level 3 facility, 50 μL of viral culture of the prototype virus was heat inactivated (99°C for 10 min), the heat-treated sample was serially diluted with standard viral culture medium, and 2 μL of serially diluted samples was added to the LAMP assay. As shown in the Figure panel C, and in Appendix Figure 1, H5N1 subtype could still be detected by the assay. However, the detection limit (9.6 pfu/reaction) was ≈1,000× less sensitive than the limit with purified RNA as an input.

## Conclusions

The RT-LAMP assay is highly specific, and its sensitivity is comparable to that of an optimized RT-PCR assay for influenza (H5N1) viruses. The detection limit is equivalent to that of a similar LAMP assay (*12*), but our assay was extensively tested by using a wide variety of influenza (H5) viruses, including recent clade 2 influenza (H5) viruses (*9*). LAMP does not require thermocyclers and gel electrophoresis. Reactions can simply be incubated in a water bath or heating block, and the results can be confirmed by direct visual inspection. Because early identification of influenza (H5) is crucial for the containment of the disease, this novel assay can provide an efficient option for the preliminary molecular detection of highly pathogenic influenza (H5) viruses in basic laboratory or clinical settings. Combined with the use of a turbidity meter, which costs much less than a real-time RT-PCR system, RT-LAMP can provide quantitative data for viral load studies. In conclusion, the LAMP assay is a promising tool for the detection of influenza (H5) viruses.

## Supplementary Material

Appendix Figure 1 Visual inspection of the positivity of LAMPreactions. Heated, treated viral culture was serially diluted and tested by the LAMP assay. Reactions were visually inspected after the incubation. Positive reactions would produce large amounts of white magnesium pyrophosphate precipitate, thereby increasing the turbidity of these reactions (+ve control and samples 1 to 3). By contrast, negative reactions (-ve control and sample 4) remained transparent after the incubation. The amount of pfu used in the tested samples is indicated.

Appendix Figure 2 Target regions

Appendix Figure 3 Reference sequence

Appendix Table Influenza A virus (H5N1) sequences used in the primer design
