# Supplementary figures and images for: Loop-Mediated Isothermal Amplification for Influenza A (H5N1) Virus
Source: Emerg Infect Dis. 2007 Jun;13(6):899–901. doi: 10.3201/eid1306.061572 (PMC2792861; doi:10.3201/eid1306.061572)

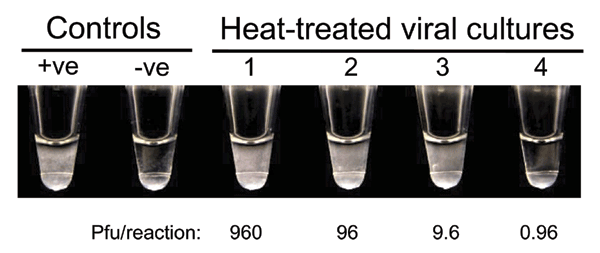

Supplement: Appendix Figure 1 — Visual inspection of the positivity of LAMPreactions. Heated, treated viral culture was serially diluted and tested by the LAMP assay. Reactions were visually inspected after the incubation. Positive reactions would produce large amounts of white magnesium pyrophosphate precipitate, thereby increasing the turbidity of these reactions (+ve control and samples 1 to 3). By contrast, negative reactions (-ve control and sample 4) remained transparent after the incubation. The amount of pfu used in the tested samples is indicated. [file 06-1572_appF1-s1.gif]

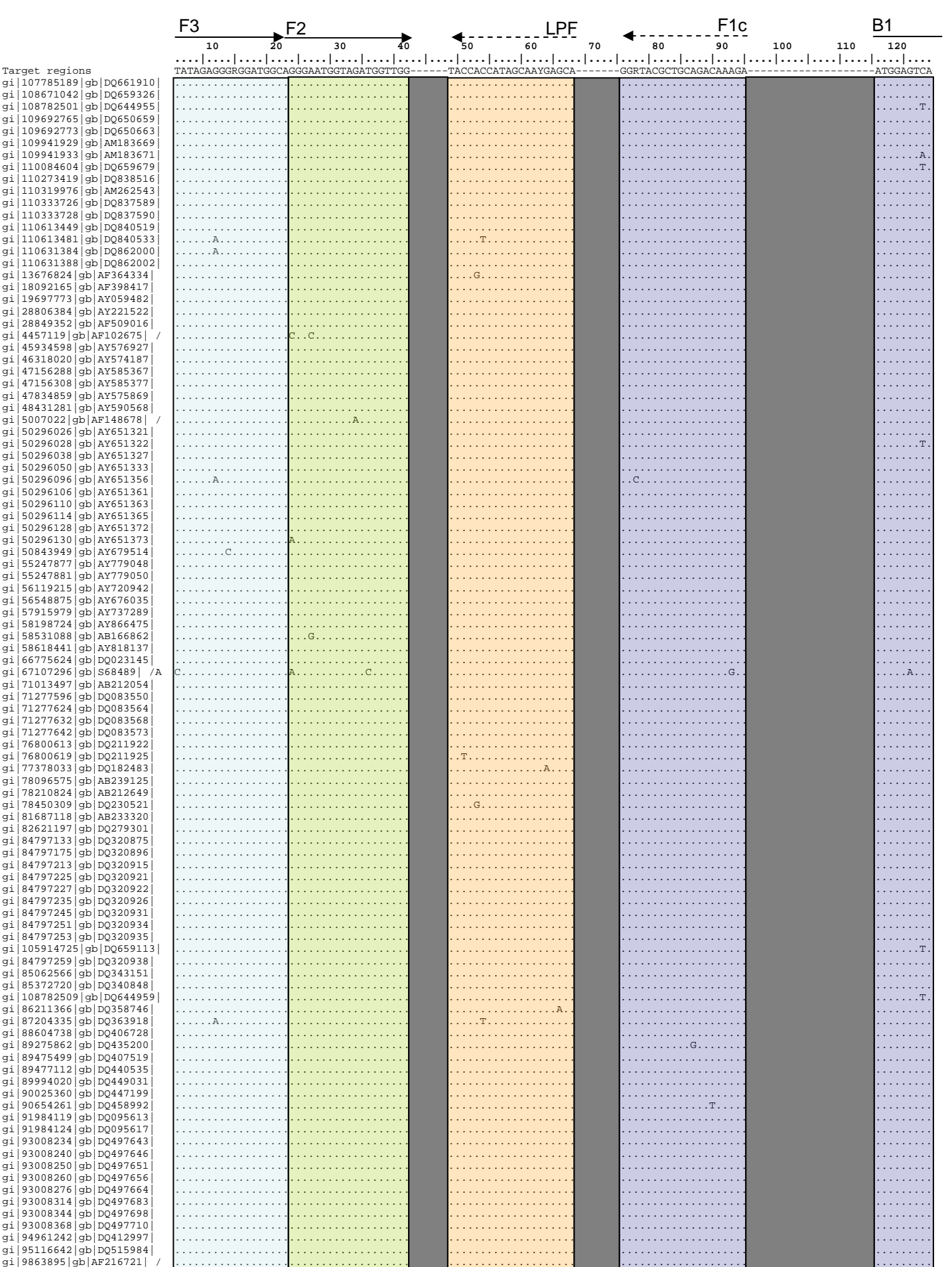

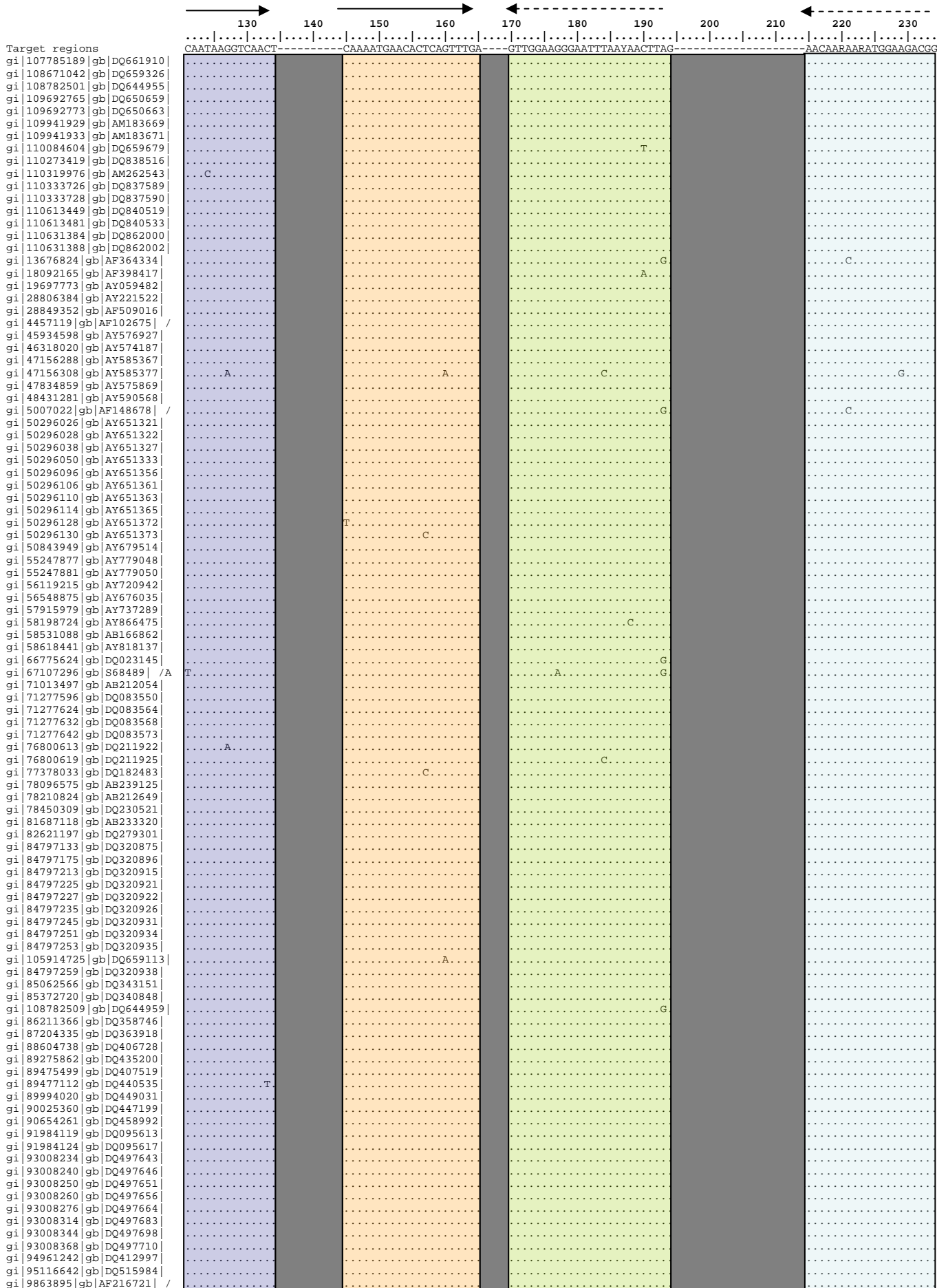

Supplement: Appendix Figure 3 — Reference sequence [file 06-1572_appF3-s3.pdf]
