# Supplementary material for: Loop-Mediated Isothermal Amplification for Influenza A (H5N1) Virus
Source: Emerg Infect Dis. 2007 Jun;13(6):899–901. doi: 10.3201/eid1306.061572 (PMC2792861; doi:10.3201/eid1306.061572)
Supplement: Appendix Figure 2 — Target regions [file 06-1572_appF2-s2.pdf]

A

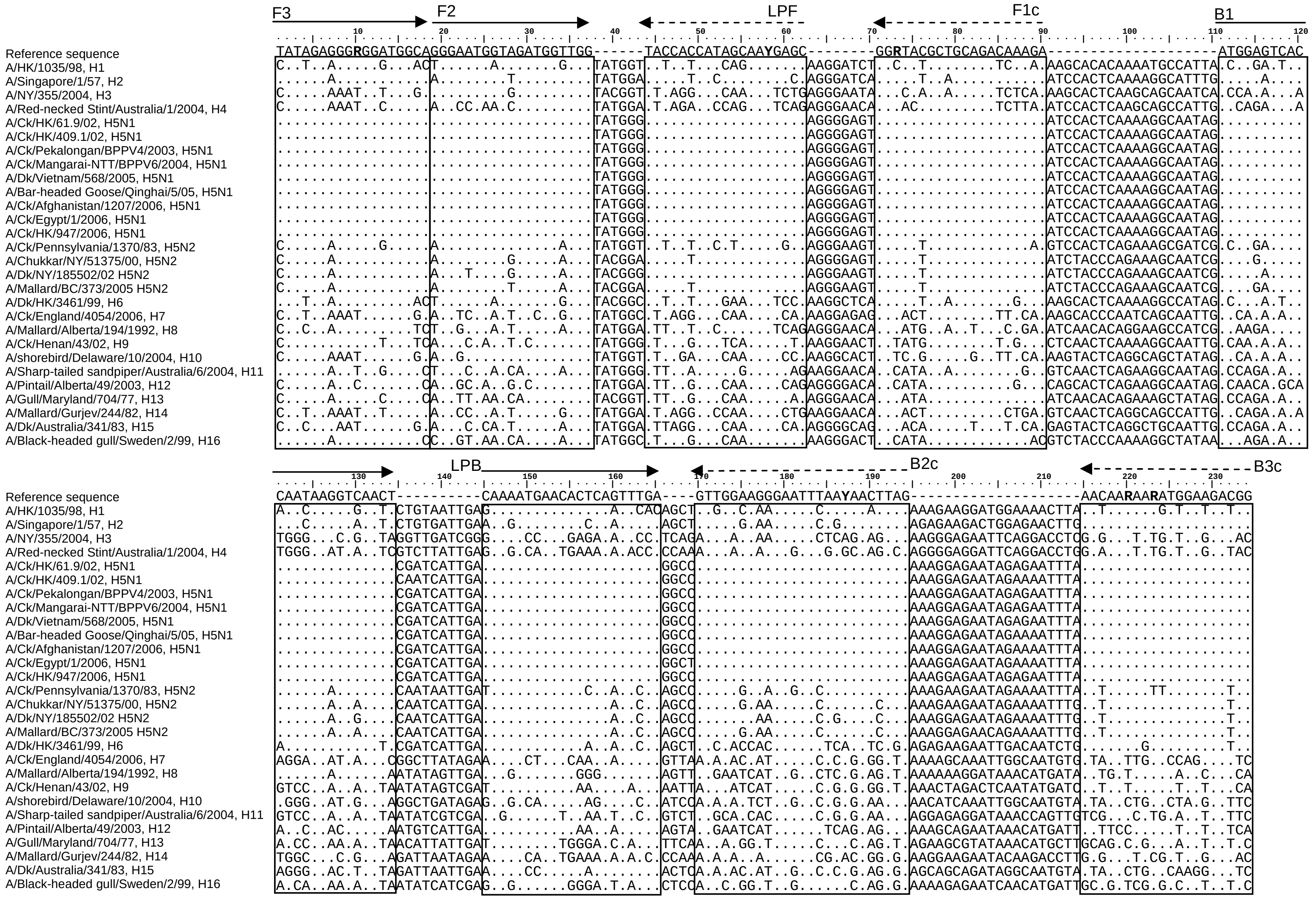

B

Primer	Sequence (5′ to 3′)
F3	TATAGAGGG**R**GGATGGCA
B3c	CCGTCTTCCAT**Y**TT**Y**TTGTT
F1P (F1c+F2)	TCTTTGTCTGCAGCGTA**Y**CCTTTTGGGAATGGTAGATGGTTGG
B1P (B1+B2c)	ATGGAGTCACCAATAAGGTCAACTTTTTCTAAGTT**R**TTAAATTCCCTTCCAAC
LPF	GCTC**R**TTGCTATGGTGGTA
LPB	CAAAATGAACACTCAGTTTGA
