# Supplementary material for: Loop-Mediated Isothermal Amplification for Influenza A (H5N1) Virus
Source: Emerg Infect Dis. 2007 Jun;13(6):899–901. doi: 10.3201/eid1306.061572 (PMC2792861; doi:10.3201/eid1306.061572)
Supplement: Appendix Table — Influenza A virus (H5N1) sequences used in the primer design [file 06-1572_appT-s4.pdf]

Appendix Table. Influenza A virus (H5N1) sequences used in the primer design

Accession number   Strains

gi|58531088|gb|AB166862| /Avian/4(HA)/H5N1/Japan/2004/// Influenza A virus (A/chicken/Yamaguchi/7/2004(H5N1)) HA gene for hemagglutinin, complete cds.
gi|58531120|gb|AB188816| /Avian/4(HA)/H5N1/Japan/2004/// Influenza A virus (A/chicken/Oita/8/2004(H5N1)) HA gene for hemagglutinin, complete cds.
gi|58531138|gb|AB188824| /Avian/4(HA)/H5N1/Japan/2004/// Influenza A virus (A/chicken/Kyoto/3/2004(H5N1)) HA gene for hemagglutinin, complete cds.
gi|58531156|gb|AB189053| /Avian/4(HA)/H5N1/Japan/2004/// Influenza A virus (A/crow/Kyoto/53/2004(H5N1)) HA gene for hemagglutinin, complete cds,.
gi|58531174|gb|AB189061| /Avian/4(HA)/H5N1/Japan/2004/// Influenza A virus (A/crow/Osaka/102/2004(H5N1)) HA gene for hemagglutinin, complete cds,.
gi|71013497|gb|AB212054| /Human/4(HA)/H5N1/Hong Kong/2003/// Influenza A virus(A/Hong Kong/213/03(H5N1)) HA gene for hemagglutinin, complete cds, MDCK isolate, embryonated chicken egg isolate.
gi|71000185|gb|AB212280| /Avian/4(HA)/H5N1/Japan/2003/// Influenza A virus (A/duck/Yokohama/aq10/2003(H5N1)) HA gene for hemagglutinin, complete cds.
gi|78210824|gb|AB212649| /Avian/4(HA)/H5N1/Japan/2004/// Influenza A virus (A/blow fly/Kyoto/93/2004(H5N1)) HA gene for hemagglutinin, complete cds.
gi|81687114|gb|AB233319| /Avian/4(HA)/H5N1/Mongolia/2005/// Influenza A virus (A/bar-headed goose/Mongolia/1/05(H5N1)) HA gene for hemagglutinin, complete cds.
gi|81687118|gb|AB233320| /Avian/4(HA)/H5N1/Mongolia/2005/// Influenza A virus (A/whooper swan/Mongolia/3/05(H5N1)) HA gene for hemagglutinin, complete cds.
gi|81687122|gb|AB233321| /Avian/4(HA)/H5N1/Mongolia/2005/// Influenza A virus (A/whooper swan/Mongolia/4/05(H5N1)) HA gene for hemagglutinin, complete cds.
gi|81687126|gb|AB233322| /Avian/4(HA)/H5N1/Mongolia/2005/// Influenza A virus (A/whooper swan/Mongolia/6/05(H5N1)) HA gene for hemagglutinin, complete cds.
gi|78096575|gb|AB239125| /Human/4(HA)/H5N1/Viet Nam/2005/// Influenza A virus (A/Hanoi/30408/2005(H5N1)) HA gene for hemagglutinin, complete cds.
gi|50296078|gb|AY651347| /Avian/4(HA)/H5N1/Hong Kong/2002/// Influenza A virus (A/Ck/HK/37.4/2002(H5N1)) hemagglutinin (HA) gene, partial cds.
gi|50296080|gb|AY651348| /Avian/4(HA)/H5N1/Hong Kong/2002/// Influenza A virus (A/SCk/HK/YU100/2002(H5N1)) hemagglutinin (HA) gene, partial cds.
gi|109809721|gb|AB263752| /Avian/4(HA)/H5N1/Mongolia/2006/// Influenza A virus (A/whooper swan/Mongolia/2/06(H5N1)) genomic RNA, segment 4, complete sequence.
gi|50296106|gb|AY651361| /Avian/4(HA)/H5N1/Hong Kong/2002/// Influenza A virus (A/tree sparrow/HK/864/2002(H5N1)) hemagglutinin (HA) gene, partial cds.
gi|50296108|gb|AY651362| /Avian/4(HA)/H5N1/Hong Kong/2004/// Influenza A virus (A/peregrine falcon/HK/D0028/2004(H5N1)) hemagglutinin (HA) gene, partial cds.
gi|50296110|gb|AY651363| /Avian/4(HA)/H5N1/China/2003/// Influenza A virus (A/Dk/HN/5806/2003(H5N1)) hemagglutinin (HA) gene, partial cds.
gi|50296120|gb|AY651368| /Avian/4(HA)/H5N1/China/2003/// Influenza A virus (A/Ck/ST/4231/2003(H5N1)) hemagglutinin (HA) gene, partial cds.
gi|50296122|gb|AY651369| /Avian/4(HA)/H5N1/China/2003/// Influenza A virus (A/Dk/YN/6255/2003(H5N1)) hemagglutinin (HA) gene, partial cds.
gi|50296124|gb|AY651370| /Avian/4(HA)/H5N1/China/2003/// Influenza A virus (A/Dk/YN/6445/2003(H5N1)) hemagglutinin (HA) gene, partial cds.
gi|50365728|gb|AY653200| /Avian/4(HA)/H5N1/China/2004/// Influenza A virus (A/chicken/Jilin/9/2004(H5N1)) segment 4, complete sequence.
gi|9863876|gb|AF216713| /Avian/4(HA)/H5N1/Hong Kong/1999/// Influenza A virus (A/Environment/Hong Kong/437-4/99 (H5N1)) hemagglutinin 5 gene, complete cds.
gi|9863895|gb|AF216721| /Avian/4(HA)/H5N1/Hong Kong/1999/// Influenza A virus (A/Environment/Hong Kong/437-6/99 (H5N1)) hemagglutinin 5 gene, complete cds.
gi|9863913|gb|AF216729| /Avian/4(HA)/H5N1/Hong Kong/1999/// Influenza A virus (A/Environment/Hong Kong/437-8/99 (H5N1)) hemagglutinin 5 gene, complete cds.
gi|9863931|gb|AF216737| /Avian/4(HA)/H5N1/Hong Kong/1999/// Influenza A virus (A/Environment/Hong Kong/437-10/99 (H5N1)) hemagglutinin 5 gene, complete cds.
gi|13676824|gb|AF364334| /Avian/4(HA)/H5N1/China/1997/// Influenza A virus (A/Goose/Guangdong/3/97(H5N1)) segment 4 hemagglutinin (HA) gene, complete cds.
gi|18092165|gb|AF398417| /Avian/4(HA)/H5N1/Hong Kong/2000/// Influenza A virus (A/Goose/Hong Kong/385.3/2000(H5N1)) hemagglutinin (HA) gene, partial cds.
gi|18092167|gb|AF398418| /Avian/4(HA)/H5N1/Hong Kong/2000/// Influenza A virus (A/Goose/Hong Kong/385.5/2000(H5N1)) hemagglutinin (HA) gene, partial cds.
gi|47600774|gb|AJ715872| /Human/4(HA)/H5N1/Viet Nam/2004/// Influenza A virus (A/Hanoi/03/2004(H5N1)) partial HA gene for hemagglutinin, genomic RNA
gi|56311401|gb|AJ867074| /Avian/4(HA)/H5N1/Viet Nam/2004/// Influenza A virus (A/Hatay/2004/(H5N1)) HA gene for hemagglutinin, genomic RNA
gi|109941929|gb|AM183669| /Avian/4(HA)/H5N1/Indonesia/2003/// Influenza A virus (A/chicken/China/1204/04(H5N1)) partial H5HA gene for hemagglutinin, genomic RNA
gi|109941931|gb|AM183670| /Avian/4(HA)/H5N1/Indonesia/2005/// Influenza A virus (A/chicken/China/1204/04(H5N1)) H5HA gene for hemagglutinin, genomic RNA
gi|109941933|gb|AM183671| /Avian/4(HA)/H5N1/China/2004/// Influenza A virus (A/chicken/Indonesia/R134/03(H5N1)) H5HA gene for hemagglutinin, genomic RNA
gi|109941935|gb|AM183672| /Avian/4(HA)/H5N1/Viet Nam/2005/// Influenza A virus (A/chicken/Indonesia/R134/03(H5N1)) H5HA gene for hemagglutinin, genomic RNA
gi|109941937|gb|AM183673| /Avian/4(HA)/H5N1/Viet Nam/2005/// Influenza A virus (A/chicken/Indonesia/R60/05(H5N1)) H5HA gene for hemagglutinin, genomic RNA
gi|109941939|gb|AM183674| /Avian/4(HA)/H5N1/Viet Nam/2005/// Influenza A virus (A/chicken/Indonesia/R60/05(H5N1)) H5HA gene for hemagglutinin, genomic RNA
gi|109941941|gb|AM183675| /Avian/4(HA)/H5N1/Viet Nam/2005/// Influenza A virus (A/chicken/Vietnam/P22/05(H5N1)) partial H5HA gene for hemagglutinin, genomic RNA
gi|109941943|gb|AM183676| /Avian/4(HA)/H5N1/Viet Nam/2005/// Influenza A virus (A/chicken/Vietnam/P22/05(H5N1)) partial H5HA gene for hemagglutinin, genomic RNA
gi|109941945|gb|AM183677| /Avian/4(HA)/H5N1/Viet Nam/2005/// Influenza A virus (A/chicken/Vietnam/P41/05(H5N1)) H5HA gene for hemagglutinin, genomic RNA
gi|54402286|gb|AY626143| /Human/4(HA)/H5N1/Thailand/2004/// Influenza A virus (A/Thailand/4(SP-528)/2004(H5N1)) hemagglutinin gene, complete cds.
gi|54299827|gb|AY627885| /Human/4(HA)/H5N1/Thailand/2004/// Influenza A virus (A/Thailand/5(KK-494)/2004(H5N1)) hemagglutinin gene, complete cds.
gi|110319972|gb|AM262541| /Avian/4(HA)/H5N1/Nigeria/2006/// Influenza A virus (A/chicken/Lagos.NIE/10.06/BA209(H5N1)) partial HA gene for hemagglutinin, genomic RNA
gi|110319974|gb|AM262542| /Avian/4(HA)/H5N1/Nigeria/2006/// Influenza A virus (A/chicken/Lagos.NIE/10.06/BA210(H5N1)) partial HA gene for hemagglutinin, genomic RNA
gi|110319976|gb|AM262543| /Avian/4(HA)/H5N1/Nigeria/2006/// Influenza A virus (A/chicken/Lagos.NIE/10.06/BA211(H5N1)) partial HA gene for hemagglutinin, genomic RNA
gi|110319978|gb|AM262546| /Avian/4(HA)/H5N1/Nigeria/2006/// Influenza A virus (A/chicken/Lagos.NIE/8.06/SO300(H5N1)) partial HA gene for hemagglutinin, genomic RNA
gi|110319980|gb|AM262547| /Avian/4(HA)/H5N1/Nigeria/2006/// Influenza A virus (A/chicken/Lagos.NIE/8.06/SO452(H5N1)) partial HA gene for hemagglutinin, genomic RNA
gi|110319982|gb|AM262553| /Avian/4(HA)/H5N1/Nigeria/2006/// Influenza A virus (A/chicken/Lagos.NIE/8.06/SO493(H5N1)) partial HA gene for hemagglutinin, genomic RNA
gi|110319984|gb|AM262572| /Avian/4(HA)/H5N1/Nigeria/2006/// Influenza A virus (A/chicken/Lagos.NIE/8.06/SO494(H5N1)) partial HA gene for hemagglutinin, genomic RNA
gi|19697757|gb|AY059474| /Avian/4(HA)/H5N1/Hong Kong/2000/// Influenza A virus (A/Goose/Hong Kong/ww26/2000(H5N1)) segment 4 hemagglutinin (HA) gene, partial cds.
gi|19697759|gb|AY059475| /Avian/4(HA)/H5N1/Hong Kong/2000/// Influenza A virus (A/Goose/Hong Kong/ww28/2000(H5N1)) segment 4 hemagglutinin (HA) gene, partial cds.
gi|19697761|gb|AY059476| /Avian/4(HA)/H5N1/Hong Kong/2000/// Influenza A virus (A/Duck/Hong Kong/ww381/2000(H5N1)) segment 4 hemagglutinin (HA) gene, partial cds.
gi|19697763|gb|AY059477| /Avian/4(HA)/H5N1/Hong Kong/2000/// Influenza A virus (A/Duck/Hong Kong/ww382/2000(H5N1)) segment 4 hemagglutinin (HA) gene, partial cds.
gi|19697765|gb|AY059478| /Avian/4(HA)/H5N1/Hong Kong/2000/// Influenza A virus (A/Duck/Hong Kong/ww461/2000(H5N1)) segment 4 hemagglutinin (HA) gene, partial cds.
gi|19697767|gb|AY059479| /Avian/4(HA)/H5N1/Hong Kong/2000/// Influenza A virus (A/Duck/Hong Kong/ww487/2000(H5N1)) segment 4 hemagglutinin (HA) gene, partial cds.
gi|19697769|gb|AY059480| /Avian/4(HA)/H5N1/Hong Kong/2000/// Influenza A virus (A/Goose/Hong Kong/ww491/2000(H5N1)) segment 4 hemagglutinin (HA) gene, partial cds.
gi|19697771|gb|AY059481| /Avian/4(HA)/H5N1/Hong Kong/2000/// Influenza A virus (A/Duck/Hong Kong/2986.1/2000(H5N1)) segment 4 hemagglutinin (HA) gene, partial cds.
gi|19697773|gb|AY059482| /Avian/4(HA)/H5N1/Hong Kong/2000/// Influenza A virus (A/Goose/Hong Kong/3014.8/2000(H5N1)) segment 4 hemagglutinin (HA) gene, partial cds.
gi|21326672|gb|AY075027| /Avian/4(HA)/H5N1/Hong Kong/2001/// Influenza A virus (A/Chicken/Hong Kong/317.5/2001(H5N1)) hemagglutinin H5 (H5) gene, complete cds.
gi|21326674|gb|AY075030| /Avian/4(HA)/H5N1/Hong Kong/2000/// Influenza A virus (A/Goose/Hong Kong/3014.5/2000(H5N1)) hemagglutinin H5 (H5) gene, complete cds.
gi|50296050|gb|AY651333| /Human/4(HA)/H5N1/Viet Nam/2004/// Influenza A virus (A/Viet Nam/1194/2004(H5N1)) hemagglutinin (HA) gene, partial cds.
gi|28805557|gb|AY221521| /Avian/4(HA)/H5N1/Hong Kong/2001/// Influenza A virus (A/Chicken/HongKong/NT873.3/01-MB(H5N1)) hemagglutinin (HA) gene, partial cds.
gi|28806384|gb|AY221522| /Avian/4(HA)/H5N1/Hong Kong/2001/// Influenza A virus (A/Chicken/HongKong/NT873.3/01(H5N1)) hemagglutinin (HA) gene, partial cds.
gi|28807284|gb|AY221523| /Avian/4(HA)/H5N1/Hong Kong/2001/// Influenza A virus (A/Chicken/HongKong/FY150/01-MB(H5N1)) hemagglutinin (HA) gene, partial cds.
gi|28807565|gb|AY221524| /Avian/4(HA)/H5N1/Hong Kong/2001/// Influenza A virus (A/Chicken/HongKong/FY150/01(H5N1)) hemagglutinin (HA) gene, partial cds.
gi|28808108|gb|AY221525| /Avian/4(HA)/H5N1/Hong Kong/2001/// Influenza A virus (A/Pheasant/HongKong/FY155/01-MB(H5N1)) hemagglutinin (HA) gene, partial cds.
gi|28808463|gb|AY221526| /Avian/4(HA)/H5N1/Hong Kong/2001/// Influenza A virus (A/Pheasant/HongKong/FY155/01(H5N1)) hemagglutinin (HA) gene, partial cds.
gi|28809438|gb|AY221527| /Avian/4(HA)/H5N1/Hong Kong/2001/// Influenza A virus (A/Chicken/HongKong/YU822.2/01-MB(H5N1)) hemagglutinin (HA) gene, partial cds.
gi|28810155|gb|AY221528| /Avian/4(HA)/H5N1/Hong Kong/2001/// Influenza A virus (A/Chicken/HongKong/YU822.2/01(H5N1)) hemagglutinin (HA) gene, partial cds.
gi|28810752|gb|AY221529| /Avian/4(HA)/H5N1/Hong Kong/2001/// Influenza A virus (A/Chicken/HongKong/YU562/01(H5N1)) hemagglutinin (HA) gene, partial cds.
gi|41207462|gb|AY518362| /Avian/4(HA)/H5N1/China/2003/// Influenza A virus (A/duck/China/E319-2/03(H5N1)) hemagglutinin subtype H5 (H5) gene, complete cds.
gi|50296072|gb|AY651344| /Avian/4(HA)/H5N1/Viet Nam/2004/// Influenza A virus (A/Dk/Viet Nam/11/2004(H5N1)) hemagglutinin (HA) gene, partial cds.
gi|50296074|gb|AY651345| /Avian/4(HA)/H5N1/Hong Kong/2002/// Influenza A virus (A/Gf/HK/38/2002(H5N1)) hemagglutinin (HA) gene, partial cds.
gi|50296076|gb|AY651346| /Avian/4(HA)/H5N1/Hong Kong/2002/// Influenza A virus (A/Ck/HK/31.2/2002(H5N1)) hemagglutinin (HA) gene, partial cds.
gi|66775624|gb|DQ023145| /Avian/4(HA)/H5N1/China/2002/// Influenza A virus (A/chicken/China/1/02(H5N1)) hemagglutinin (HA) mRNA, complete cds.
gi|67527197|gb|DQ076201| /Avian/4(HA)/H5N1/Thailand/2004/// Influenza A virus (A/Ck/Thailand/73/2004(H5N1)) hemagglutinin (HA) gene, complete cds.
gi|50296082|gb|AY651349| /Avian/4(HA)/H5N1/Hong Kong/2002/// Influenza A virus (A/Ck/HK/YU22/2002(H5N1)) hemagglutinin (HA) gene, partial cds.
gi|50296084|gb|AY651350| /Avian/4(HA)/H5N1/Hong Kong/2002/// Influenza A virus (A/Ck/HK/3176.3/2002(H5N1)) hemagglutinin (HA) gene, partial cds.
gi|50296086|gb|AY651351| /Avian/4(HA)/H5N1/Hong Kong/2002/// Influenza A virus (A/Ck/HK/3169.1/2002(H5N1)) hemagglutinin (HA) gene, partial cds.
gi|50296088|gb|AY651352| /Avian/4(HA)/H5N1/China/2002/// Influenza A virus (A/teal/China/2978.1/2002(H5N1)) hemagglutinin (HA) gene, partial cds.
gi|50296090|gb|AY651353| /Avian/4(HA)/H5N1/Hong Kong/2003/// Influenza A virus (A/Ck/HK/2133.1/2003(H5N1)) hemagglutinin (HA) gene, partial cds.
gi|50296092|gb|AY651354| /Avian/4(HA)/H5N1/Hong Kong/2003/// Influenza A virus (A/Ck/HK/NT93/2003(H5N1)) hemagglutinin (HA) gene, partial cds.
gi|50296094|gb|AY651355| /Avian/4(HA)/H5N1/Hong Kong/2003/// Influenza A virus (A/Ck/HK/WF157/2003(H5N1)) hemagglutinin (HA) gene, partial cds.
gi|50296096|gb|AY651356| /Avian/4(HA)/H5N1/Hong Kong/2003/// Influenza A virus (A/Ck/HK/SSP141/2003(H5N1)) hemagglutinin (HA) gene, partial cds.
gi|50296098|gb|AY651357| /Avian/4(HA)/H5N1/Hong Kong/2003/// Influenza A virus (A/Ck/HK/FY157/2003(H5N1)) hemagglutinin (HA) gene, partial cds.
gi|50296100|gb|AY651358| /Avian/4(HA)/H5N1/Hong Kong/2003/// Influenza A virus (A/Ck/HK/YU324/2003(H5N1)) hemagglutinin (HA) gene, partial cds.
gi|50296102|gb|AY651359| /Avian/4(HA)/H5N1/Hong Kong/2002/// Influenza A virus (A/grey heron/HK/861.1/2002(H5N1)) hemagglutinin (HA) gene, partial cds.

gi|50296104|gb|AY651360| /Avian/4(HA)/H5N1/Hong Kong/2002/// Influenza A virus (A/feral pigeon/HK/862.7/2002(H5N1)) hemagglutinin (HA) gene, partial cds.
gi|71277618|gb|DQ083561| /Avian/4(HA)/H5N1/Thailand/2004/// Influenza A virus (A/chicken/Nakhon Sawan/Thailand/CU-13/04(H5N1)) hemagglutinin gene, partial cds.
gi|71277620|gb|DQ083562| /Avian/4(HA)/H5N1/Thailand/2004/// Influenza A virus (A/chicken/Nakhon Pathom/Thailand/CU-14/04(H5N1)) hemagglutinin gene, partial cds.
gi|71277622|gb|DQ083563| /Avian/4(HA)/H5N1/Thailand/2004/// Influenza A virus (A/crow/Bangkok/Thailand/CU-15/04(H5N1)) hemagglutinin gene, complete cds.
gi|50296112|gb|AY651364| /Avian/4(HA)/H5N1/China/2004/// Influenza A virus (A/Dk/HN/303/2004(H5N1)) hemagglutinin (HA) gene, partial cds.
gi|50296114|gb|AY651365| /Avian/4(HA)/H5N1/China/2004/// Influenza A virus (A/Dk/HN/101/2004(H5N1)) hemagglutinin (HA) gene, partial cds.
gi|50296116|gb|AY651366| /Avian/4(HA)/H5N1/China/2004/// Influenza A virus (A/Ph/ST/44/2004(H5N1)) hemagglutinin (HA) gene, partial cds.
gi|50296118|gb|AY651367| /Avian/4(HA)/H5N1/China/2003/// Influenza A virus (A/Dk/ST/4003/2003(H5N1)) hemagglutinin (HA) gene, partial cds.
gi|71277632|gb|DQ083568| /Avian/4(HA)/H5N1/Thailand/2004/// Influenza A virus (A/chicken/Bangkok/Thailand/CU-20/04(H5N1)) hemagglutinin gene, partial cds.
gi|71277634|gb|DQ083569| /Avian/4(HA)/H5N1/Thailand/2004/// Influenza A virus (A/chicken/Ayutthaya/Thailand/CU-24/04(H5N1)) hemagglutinin gene, partial cds.
gi|71277636|gb|DQ083570| /Avian/4(HA)/H5N1/Thailand/2004/// Influenza A virus (A/crow/Bangkok/Thailand/CU-25/04(H5N1)) hemagglutinin gene, partial cds.
gi|50296126|gb|AY651371| /Avian/4(HA)/H5N1/China/2004/// Influenza A virus (A/Ck/YN/374/2004(H5N1)) hemagglutinin (HA) gene, partial cds.
gi|50296128|gb|AY651372| /Avian/4(HA)/H5N1/China/2004/// Influenza A virus (A/Ck/YN/115/2004(H5N1)) hemagglutinin (HA) gene, partial cds.
gi|50296130|gb|AY651373| /Avian/4(HA)/H5N1/Hong Kong/2003/// Influenza A virus (A/black headed gull/HK/12.1/2003(H5N1)) hemagglutinin (HA) gene, partial cds.
gi|71277644|gb|DQ083574| /Avian/4(HA)/H5N1/Thailand/2004/// Influenza A virus (A/ostrich/Samut Prakan/Thailand/CU-31/04(H5N1)) hemagglutinin gene, partial cds.
gi|56548871|gb|AY676033| /Avian/4(HA)/H5N1/Hong Kong/2002/// Influenza A virus (A/duck/Hong Kong/821/02(H5N1)) hemagglutinin (HA) gene, complete cds.
gi|56548873|gb|AY676034| /Avian/4(HA)/H5N1/Hong Kong/2003/// Influenza A virus (A/egret/Hong Kong/757.2/03(H5N1)) hemagglutinin (HA) gene, complete cds.
gi|56548875|gb|AY676035| /Avian/4(HA)/H5N1/South Korea/2003/// Influenza A virus (A/chicken/Korea/ES/03(H5N1)) hemagglutinin (HA) gene, complete cds.
gi|56548877|gb|AY676036| /Avian/4(HA)/H5N1/South Korea/2003/// Influenza A virus (A/duck/Korea/ESD1/03(H5N1)) hemagglutinin (HA) gene, complete cds.
gi|50843949|gb|AY679514| /Human/4(HA)/H5N1/Thailand/2004/// Influenza A virus (A/Thailand/LFPN-2004/2004(H5N1)) hemagglutinin mRNA, complete cds.
gi|50956627|gb|AY684706| /Avian/4(HA)/H5N1/China/2004/// Influenza A virus (A/chicken/Hubei/327/2004(H5N1)) hemagglutinin (HA) gene, complete cds.
gi|46578137|gb|AY555150| /Human/4(HA)/H5N1/Thailand/2004/// Influenza A virus (A/Thailand/1(KAN-1)/2004(H5N1)) hemagglutinin gene, partial cds.
gi|45453833|gb|AY555153| /Human/4(HA)/H5N1/Thailand/2004/// Influenza A virus (A/Thailand/2(SP-33)/2004(H5N1)) hemagglutinin gene, complete cds.
gi|46318020|gb|AY574187| /Avian/4(HA)/H5N1/Viet Nam/2004/// Influenza A virus (A/chicken/Vietnam/HD1/2004(H5N1)) hemagglutinin gene, partial cds.
gi|46318026|gb|AY574190| /Avian/4(HA)/H5N1/Viet Nam/2004/// Influenza A virus (A/chicken/Vietnam/HD2/2004(H5N1)) hemagglutinin gene, partial cds.
gi|47834859|gb|AY575869| /Human/4(HA)/H5N1/Hong Kong/2003/// Influenza A virus (A/HK/212/03 (H5N1)) hemagglutinin (HA) gene, partial cds.
gi|47834861|gb|AY575870| /Human/4(HA)/H5N1/Hong Kong/2003/// Influenza A virus (A/HK/213/03 (H5N1)) hemagglutinin (HA) gene, partial cds.
gi|47834863|gb|AY575871| /Avian/4(HA)/H5N1/Hong Kong/2002/// Influenza A virus (A/Gs/HK/739.2/02 (H5N1)) hemagglutinin (HA) gene, partial cds.
gi|47834865|gb|AY575872| /Avian/4(HA)/H5N1/Hong Kong/2002/// Influenza A virus (A/Eg/HK/757.3/02 (H5N1)) hemagglutinin (HA) gene, partial cds.
gi|47834875|gb|AY575873| /Avian/4(HA)/H5N1/Hong Kong/2002/// Influenza A virus (A/G.H/HK/793.1/02 (H5N1)) hemagglutinin (HA) gene, partial cds.
gi|47834877|gb|AY575874| /Avian/4(HA)/H5N1/Hong Kong/2002/// Influenza A virus (A/Dk/HK/821/02 (H5N1)) hemagglutinin (HA) gene, partial cds.
gi|91984124|gb|DQ095617| /Avian/4(HA)/H5N1/China/2005/// Influenza A virus (A/Bar-headed Goose/Qinghai/5/05(H5N1)) hemagglutinin (HA) gene, partial cds.
gi|47834881|gb|AY575876| /Avian/4(HA)/H5N1/Hong Kong/2002/// Influenza A virus (A/Ck/HK/61.9/02 (H5N1)) hemagglutinin (HA) gene, partial cds.
gi|47834883|gb|AY575877| /Avian/4(HA)/H5N1/Hong Kong/2002/// Influenza A virus (A/Ck/HK/YU777/02 (H5N1)) hemagglutinin (HA) gene, partial cds.
gi|57916028|gb|AY737296| /Avian/4(HA)/H5N1/China/2004/// Influenza A virus (A/chicken/Guangdong/178/04(H5N1)) segment 4, complete sequence.
gi|57916076|gb|AY737304| /Avian/4(HA)/H5N1/China/2004/// Influenza A virus (A/duck/Guangdong/173/04(H5N1)) segment 4, complete sequence.
gi|47834889|gb|AY575880| /Avian/4(HA)/H5N1/Hong Kong/2002/// Influenza A virus (A/Ph/HK/675.14/02 (H5N1)) hemagglutinin (HA) gene, partial cds.
gi|45934598|gb|AY576927| /Avian/4(HA)/H5N1/Viet Nam/2004/// Influenza A virus (A/chicken/Vietnam/CM/2004(H5N1)) segment 4 hemagglutinin gene, partial cds.
gi|45934604|gb|AY576930| /Avian/4(HA)/H5N1/Viet Nam/2004/// Influenza A virus (A/muscovy duck/Vietnam/MdGL/2004(H5N1)) segment 4 hemagglutinin gene, partial cds.
gi|46578415|gb|AY577314| /Human/4(HA)/H5N1/Thailand/2004/// Influenza A virus (A/Thailand/3(SP-83)/2004(H5N1)) hemagglutinin gene, complete cds.
gi|47156268|gb|AY585357| /Avian/4(HA)/H5N1/China/2002/// Influenza A virus (A/duck/Fujian/01/2002(H5N1)) hemagglutinin (HA) mRNA, complete cds.
gi|47156270|gb|AY585358| /Avian/4(HA)/H5N1/China/2002/// Influenza A virus (A/duck/Fujian/13/2002(H5N1)) hemagglutinin (HA) mRNA, complete cds.
gi|47156272|gb|AY585359| /Avian/4(HA)/H5N1/China/2000/// Influenza A virus (A/duck/Fujian/19/2000(H5N1)) hemagglutinin (HA) mRNA, complete cds.
gi|47156274|gb|AY585360| /Avian/4(HA)/H5N1/China/2001/// Influenza A virus (A/duck/Guangdong/01/2001(H5N1)) hemagglutinin (HA) mRNA, complete cds.
gi|47156276|gb|AY585361| /Avian/4(HA)/H5N1/China/2000/// Influenza A virus (A/duck/Guangdong/12/2000(H5N1)) hemagglutinin (HA) mRNA, complete cds.
gi|55247877|gb|AY779048| /Avian/4(HA)/H5N1/Thailand/2004/// Influenza A virus (A/duck/Thailand/CU-2/2004 (H5N1)) hemagglutinin gene, partial cds.
gi|47156280|gb|AY585363| /Avian/4(HA)/H5N1/China/1999/// Influenza A virus (A/duck/Guangxi/07/1999(H5N1)) hemagglutinin (HA) mRNA, complete cds.
gi|47156282|gb|AY585364| /Avian/4(HA)/H5N1/China/2001/// Influenza A virus (A/duck/Guangxi/22/2001(H5N1)) hemagglutinin (HA) mRNA, complete cds.
gi|47156284|gb|AY585365| /Avian/4(HA)/H5N1/China/2001/// Influenza A virus (A/duck/Guangxi/35/2001(H5N1)) hemagglutinin (HA) mRNA, complete cds.
gi|47156286|gb|AY585366| /Avian/4(HA)/H5N1/China/2002/// Influenza A virus (A/duck/Guangxi/53/2002(H5N1)) hemagglutinin (HA) mRNA, complete cds.
gi|47156288|gb|AY585367| /Avian/4(HA)/H5N1/China/2001/// Influenza A virus (A/duck/Shanghai/13/2001(H5N1)) hemagglutinin (HA) mRNA, complete cds.
gi|47156290|gb|AY585368| /Avian/4(HA)/H5N1/China/2002/// Influenza A virus (A/duck/Shanghai/35/2002(H5N1)) hemagglutinin (HA) mRNA, complete cds.
gi|47156292|gb|AY585369| /Avian/4(HA)/H5N1/China/2002/// Influenza A virus (A/duck/Shanghai/37/2002(H5N1)) hemagglutinin (HA) mRNA, complete cds.
gi|47156294|gb|AY585370| /Avian/4(HA)/H5N1/China/2001/// Influenza A virus (A/duck/Shanghai/08/2001(H5N1)) hemagglutinin (HA) mRNA, complete cds.
gi|47156296|gb|AY585371| /Avian/4(HA)/H5N1/China/2000/// Influenza A virus (A/duck/Zhejiang/11/2000(H5N1)) hemagglutinin (HA) mRNA, complete cds.
gi|47156298|gb|AY585372| /Avian/4(HA)/H5N1/China/2001/// Influenza A virus (A/duck/Fujian/17/2001(H5N1)) hemagglutinin (HA) mRNA, complete cds.
gi|47156300|gb|AY585373| /Avian/4(HA)/H5N1/China/2000/// Influenza A virus (A/duck/Guangdong/07/2000(H5N1)) hemagglutinin (HA) mRNA, complete cds.
gi|47156302|gb|AY585374| /Avian/4(HA)/H5N1/China/2000/// Influenza A virus (A/duck/Guangdong/40/2000(H5N1)) hemagglutinin (HA) mRNA, complete cds.
gi|47156304|gb|AY585375| /Avian/4(HA)/H5N1/China/2001/// Influenza A virus (A/duck/Guangxi/50/2001(H5N1)) hemagglutinin (HA) mRNA, complete cds.
gi|47156306|gb|AY585376| /Avian/4(HA)/H5N1/China/2001/// Influenza A virus (A/duck/Shanghai/38/2001(H5N1)) hemagglutinin (HA) mRNA, complete cds.
gi|47156308|gb|AY585377| /Avian/4(HA)/H5N1/China/2000/// Influenza A virus (A/duck/Zhejiang/52/2000(H5N1)) hemagglutinin (HA) mRNA, complete cds.
gi|46361433|gb|AY590563| /Avian/4(HA)/H5N1/Thailand/2004/// Influenza A virus (A/chicken/Thailand/CU-K1/2004(H5N1)) hemagglutinin gene, partial cds.
gi|48431281|gb|AY590568| /Avian/4(HA)/H5N1/Thailand/2004/// Influenza A virus (A/chicken/Nakorn-Patom/Thailand/CU-K2/2004(H5N1)) hemagglutinin gene, partial cds.
gi|46361437|gb|AY590569| /Avian/4(HA)/H5N1/Thailand/2004/// Influenza A virus (A/kalij pheasant/Thailand/CU-4/2004(H5N1)) hemagglutinin gene, partial cds.
gi|46361439|gb|AY590570| /Avian/4(HA)/H5N1/Thailand/2004/// Influenza A virus (A/white peafowl/Thailand/CU-11/2004(H5N1)) hemagglutinin gene, partial cds.
gi|62466140|gb|AY972540| /Tiger/4(HA)/H5N1/Thailand/2004/// Influenza A virus (A/tiger/Thailand/CU-T5/04(H5N1)) hemagglutinin (HA) gene, partial cds.
gi|62466142|gb|AY972541| /Tiger/4(HA)/H5N1/Thailand/2004/// Influenza A virus (A/tiger/Thailand/CU-T6/04(H5N1)) hemagglutinin (HA) gene, complete cds.
gi|62466144|gb|AY972542| /Tiger/4(HA)/H5N1/Thailand/2004/// Influenza A virus (A/tiger/Thailand/CU-T8/04(H5N1)) hemagglutinin (HA) gene, partial cds.
gi|62910858|gb|DQ003215| /Avian/4(HA)/H5N1/China/2001/// Influenza A virus (A/chicken/Jiande/1218/2001(H5N1)) hemagglutinin (HA) gene, complete cds.
gi|76800619|gb|DQ211925| /Avian/4(HA)/H5N1/China/2003/// Influenza A virus (A/chicken/luohuo/3/03(H5N1)) hemagglutinin (HA) gene, complete cds.
gi|77641488|gb|DQ212792| /Avian/4(HA)/H5N1/Russia/2005/// Influenza A virus (A/goose/Novosibirsk/4/2005(H5N1)) hemagglutinin (HA) gene, partial cds.
gi|46361445|gb|AY590577| /Avian/4(HA)/H5N1/Thailand/2004/// Influenza A virus (A/openbill/Thailand/CU-2/2004(H5N1)) hemagglutinin gene, partial cds.
gi|47716772|gb|AY609312| /Avian/4(HA)/H5N1/China/2004/// Influenza A virus (A/chicken/Guangdong/174/04(H5N1)) segment 4, complete sequence.
gi|50952819|gb|AY623430| /Avian/4(HA)/H5N1/China/2004/// Influenza A virus (A/chicken/Yichang/lung-1/04(H5N1)) hemagglutinin gene, partial cds.
gi|78146083|gb|DQ231241| /Avian/4(HA)/H5N1/Russia/2005/// Influenza A virus (A/chicken/Suzdalka/Nov-12/05(H5N1)) hemagglutinin (HA) gene, partial cds.
gi|78146106|gb|DQ231242| /Avian/4(HA)/H5N1/Russia/2005/// Influenza A virus (A/chicken/Suzdalka/Nov-11/05(H5N1)) hemagglutinin (HA) gene, partial cds.
gi|49356775|gb|AY639405| /Avian/4(HA)/H5N1/China/2004/// Influenza A virus (A/goose/China/F3/2004(H5N1)) hemagglutinin (H5) gene, complete cds.
gi|50083230|gb|AY646167| /Tiger/4(HA)/H5N1/Thailand/2004/// Influenza A virus (A/tiger/Suphanburi/Thailand/Ti-1/04(H5N1)) hemagglutinin gene, complete cds.
gi|50083246|gb|AY646175| /Leopard/4(HA)/H5N1/Thailand/2004/// Influenza A virus (A/leopard/Suphanburi/Thailand/Leo-1/04(H5N1)) hemagglutinin gene, complete cds.
gi|50261904|gb|AY646424| /Swine/4(HA)/H5N1/China/2003/// Influenza virus A (A/swine/Shandong/2/03(H5N1)) hemagglutinin (HA) gene, complete cds.
gi|55793690|gb|AY649382| /Avian/4(HA)/H5N1/Thailand/2004/// Influenza A virus (A/chicken/Thailand/CH-2/2004(H5N1)) hemagglutinin gene, complete cds.
gi|50296024|gb|AY651320| /Avian/4(HA)/H5N1/Indonesia/2003/// Influenza A virus (A/Ck/Indonesia/PA/2003(H5N1)) hemagglutinin (HA) gene, partial cds.
gi|50296026|gb|AY651321| /Avian/4(HA)/H5N1/Indonesia/2003/// Influenza A virus (A/Ck/Indonesia/BL/2003(H5N1)) hemagglutinin (HA) gene, partial cds.
gi|50296028|gb|AY651322| /Avian/4(HA)/H5N1/Indonesia/2004/// Influenza A virus (A/Dk/Indonesia/MS/2004(H5N1)) hemagglutinin (HA) gene, partial cds.
gi|50296030|gb|AY651323| /Avian/4(HA)/H5N1/Indonesia/2003/// Influenza A virus (A/Ck/Indonesia/2A/2003(H5N1)) hemagglutinin (HA) gene, partial cds.
gi|50296032|gb|AY651324| /Avian/4(HA)/H5N1/Indonesia/2004/// Influenza A virus (A/Ck/Indonesia/4/2004(H5N1)) hemagglutinin (HA) gene, partial cds.
gi|50296034|gb|AY651325| /Avian/4(HA)/H5N1/Indonesia/2004/// Influenza A virus (A/Ck/Indonesia/5/2004(H5N1)) hemagglutinin (HA) gene, partial cds.
gi|50296036|gb|AY651326| /Avian/4(HA)/H5N1/Thailand/2004/// Influenza A virus (A/Ck/Thailand/1/2004(H5N1)) hemagglutinin (HA) gene, partial cds.
gi|21359659|gb|AF468837| /Avian/4(HA)/H5N1/China/2001/// Influenza A virus (A/Duck/Anyang/AVL-1/2001(H5N1)) hemagglutinin H5 (H5) gene, complete cds.
gi|28849352|gb|AF509016| /Avian/4(HA)/H5N1/Hong Kong/2001/// Influenza A virus (A/Chicken/Hong Kong/FY77/01 (H5N1)) hemagglutinin (HA) gene, partial cds.
gi|28849354|gb|AF509017| /Avian/4(HA)/H5N1/Hong Kong/2001/// Influenza A virus (A/Chicken/Hong Kong/YU562/01 (H5N1)) hemagglutinin (HA) gene, partial cds.
gi|28849356|gb|AF509018| /Avian/4(HA)/H5N1/Hong Kong/2001/// Influenza A virus (A/Chicken/Hong Kong/YU563/01 (H5N1)) hemagglutinin (HA) gene, partial cds.
gi|28849358|gb|AF509019| /Avian/4(HA)/H5N1/Hong Kong/2001/// Influenza A virus (A/Chicken/Hong Kong/FY150/01 (H5N1)) hemagglutinin (HA) gene, partial cds.

gi|28849360|gb|AF509020| /Avian/4(HA)/H5N1/Hong Kong/2001/// Influenza A virus (A/Pheasant/Hong Kong/FY155/01 (H5N1)) hemagglutinin (HA) gene, partial cds.
gi|28849362|gb|AF509021| /Avian/4(HA)/H5N1/Hong Kong/2001/// Influenza A virus (A/Silky Chicken/Hong Kong/SF189/01 (H5N1)) hemagglutinin (HA) gene, partial cds.
gi|28849364|gb|AF509022| /Avian/4(HA)/H5N1/Hong Kong/2001/// Influenza A virus (A/Quail/Hong Kong/SF203/01 (H5N1)) hemagglutinin (HA) gene, partial cds.
gi|28849366|gb|AF509023| /Avian/4(HA)/H5N1/Hong Kong/2001/// Influenza A virus (A/Pigeon/Hong Kong/SF215/01 (H5N1)) hemagglutinin (HA) gene, partial cds.
gi|28849368|gb|AF509024| /Avian/4(HA)/H5N1/Hong Kong/2001/// Influenza A virus (A/Chicken/Hong Kong/SF219/01 (H5N1)) hemagglutinin (HA) gene, partial cds.
gi|28849370|gb|AF509025| /Avian/4(HA)/H5N1/Hong Kong/2001/// Influenza A virus (A/Chicken/Hong Kong/715.5/01 (H5N1)) hemagglutinin (HA) gene, partial cds.
gi|28849372|gb|AF509026| /Avian/4(HA)/H5N1/Hong Kong/2001/// Influenza A virus (A/Chicken/Hong Kong/822.1/01 (H5N1)) hemagglutinin (HA) gene, partial cds.
gi|28849374|gb|AF509027| /Avian/4(HA)/H5N1/Hong Kong/2001/// Influenza A virus (A/Chicken/Hong Kong/829.2/01 (H5N1)) hemagglutinin (HA) gene, partial cds.
gi|28849376|gb|AF509028| /Avian/4(HA)/H5N1/Hong Kong/2001/// Influenza A virus (A/Chicken/Hong Kong/830.2/01 (H5N1)) hemagglutinin (HA) gene, partial cds.
gi|28849378|gb|AF509029| /Avian/4(HA)/H5N1/Hong Kong/2001/// Influenza A virus (A/Chicken/Hong Kong/858.3/01 (H5N1)) hemagglutinin (HA) gene, partial cds.
gi|28849380|gb|AF509030| /Avian/4(HA)/H5N1/Hong Kong/2001/// Influenza A virus (A/Chicken/Hong Kong/867.1/01 (H5N1)) hemagglutinin (HA) gene, partial cds.
gi|28849382|gb|AF509031| /Avian/4(HA)/H5N1/Hong Kong/2001/// Influenza A virus (A/Chicken/Hong Kong/879.1/01 (H5N1)) hemagglutinin (HA) gene, partial cds.
gi|28849384|gb|AF509032| /Avian/4(HA)/H5N1/Hong Kong/2001/// Influenza A virus (A/Chicken/Hong Kong/873.3/01 (H5N1)) hemagglutinin (HA) gene, partial cds.
gi|28849386|gb|AF509033| /Avian/4(HA)/H5N1/Hong Kong/2001/// Influenza A virus (A/Chicken/Hong Kong/876.1/01 (H5N1)) hemagglutinin (HA) gene, partial cds.
gi|28849388|gb|AF509034| /Avian/4(HA)/H5N1/Hong Kong/2001/// Influenza A virus (A/Chicken/Hong Kong/891.1/01 (H5N1)) hemagglutinin (HA) gene, partial cds.
gi|28849390|gb|AF509035| /Avian/4(HA)/H5N1/Hong Kong/2001/// Influenza A virus (A/Chicken/Hong Kong/893.2/01 (H5N1)) hemagglutinin (HA) gene, partial cds.
gi|28849392|gb|AF509036| /Avian/4(HA)/H5N1/Hong Kong/2001/// Influenza A virus (A/Goose/Hong Kong/76.1/01 (H5N1)) hemagglutinin (HA) gene, partial cds.
gi|28849394|gb|AF509037| /Avian/4(HA)/H5N1/Hong Kong/2001/// Influenza A virus (A/Goose/Hong Kong/ww100/01 (H5N1)) hemagglutinin (HA) gene, partial cds.
gi|28849396|gb|AF509038| /Avian/4(HA)/H5N1/Hong Kong/2001/// Influenza A virus (A/Duck/Hong Kong/573.4/01 (H5N1)) hemagglutinin (HA) gene, partial cds.
gi|28849398|gb|AF509039| /Avian/4(HA)/H5N1/Hong Kong/2001/// Influenza A virus (A/Duck/Hong Kong/646.3/01 (H5N1)) hemagglutinin (HA) gene, partial cds.
gi|50296038|gb|AY651327| /Avian/4(HA)/H5N1/Thailand/2004/// Influenza A virus (A/Ck/Thailand/73/2004(H5N1)) hemagglutinin (HA) gene, partial cds.
gi|50296040|gb|AY651328| /Avian/4(HA)/H5N1/Thailand/2004/// Influenza A virus (A/Ck/Thailand/9.1/2004(H5N1)) hemagglutinin (HA) gene, partial cds.
gi|50296042|gb|AY651329| /Avian/4(HA)/H5N1/Thailand/2004/// Influenza A virus (A/Qa/Thailand/57/2004(H5N1)) hemagglutinin (HA) gene, partial cds.
gi|50296044|gb|AY651330| /Avian/4(HA)/H5N1/Thailand/2004/// Influenza A virus (A/bird/Thailand/3.1/2004(H5N1)) hemagglutinin (HA) gene, partial cds.
gi|50296046|gb|AY651331| /Avian/4(HA)/H5N1/Thailand/2004/// Influenza A virus (A/Dk/Thailand/71.1/2004(H5N1)) hemagglutinin (HA) gene, partial cds.
gi|50296048|gb|AY651332| /Avian/4(HA)/H5N1/Thailand/2004/// Influenza A virus (A/Gs/Thailand/79/2004(H5N1)) hemagglutinin (HA) gene, partial cds.
gi|84797151|gb|DQ320884| /Avian/4(HA)/H5N1/China/2004/// Influenza A virus (A/duck/Guangxi/1378/2004(H5N1)) hemagglutinin (HA) gene, partial cds.
gi|50296054|gb|AY651335| /Human/4(HA)/H5N1/Viet Nam/2004/// Influenza A virus (A/Viet Nam/3046/2004(H5N1)) hemagglutinin (HA) gene, partial cds.
gi|50296056|gb|AY651336| /Human/4(HA)/H5N1/Viet Nam/2004/// Influenza A virus (A/Viet Nam/3062/2004(H5N1)) hemagglutinin (HA) gene, partial cds.
gi|50296058|gb|AY651337| /Avian/4(HA)/H5N1/Viet Nam/2004/// Influenza A virus (A/Ck/Viet Nam/33/2004(H5N1)) hemagglutinin (HA) gene, partial cds.
gi|50296060|gb|AY651338| /Avian/4(HA)/H5N1/Viet Nam/2004/// Influenza A virus (A/Ck/Viet Nam/35/2004(H5N1)) hemagglutinin (HA) gene, partial cds.
gi|50296062|gb|AY651339| /Avian/4(HA)/H5N1/Viet Nam/2004/// Influenza A virus (A/Ck/Viet Nam/36/2004(H5N1)) hemagglutinin (HA) gene, partial cds.
gi|50296064|gb|AY651340| /Avian/4(HA)/H5N1/Viet Nam/2004/// Influenza A virus (A/Ck/Viet Nam/37/2004(H5N1)) hemagglutinin (HA) gene, partial cds.
gi|50296066|gb|AY651341| /Avian/4(HA)/H5N1/Viet Nam/2004/// Influenza A virus (A/Ck/Viet Nam/38/2004(H5N1)) hemagglutinin (HA) gene, partial cds.
gi|50296068|gb|AY651342| /Avian/4(HA)/H5N1/Viet Nam/2004/// Influenza A virus (A/Ck/Viet Nam/39/2004(H5N1)) hemagglutinin (HA) gene, partial cds.
gi|50296070|gb|AY651343| /Avian/4(HA)/H5N1/Viet Nam/2004/// Influenza A virus (A/Ck/Viet Nam/C57/2004(H5N1)) hemagglutinin (HA) gene, partial cds.
gi|84797173|gb|DQ320895| /Avian/4(HA)/H5N1/China/2004/// Influenza A virus (A/chicken/Guangxi/2461/2004(H5N1)) hemagglutinin (HA) gene, partial cds.
gi|84797175|gb|DQ320896| /Avian/4(HA)/H5N1/China/2005/// Influenza A virus (A/goose/Guangxi/345/2005(H5N1)) hemagglutinin (HA) gene, partial cds.
gi|84797177|gb|DQ320897| /Avian/4(HA)/H5N1/China/2005/// Influenza A virus (A/quail/Guangxi/575/2005(H5N1)) hemagglutinin (HA) gene, partial cds.
gi|84797179|gb|DQ320898| /Avian/4(HA)/H5N1/China/2005/// Influenza A virus (A/chicken/Guangxi/604/2005(H5N1)) hemagglutinin (HA) gene, partial cds.
gi|84797181|gb|DQ320899| /Avian/4(HA)/H5N1/China/2005/// Influenza A virus (A/duck/Guangxi/793/2005(H5N1)) hemagglutinin (HA) gene, partial cds.
gi|67649668|gb|DQ080022| /Avian/4(HA)/H5N1/China/2004/// Influenza A virus (A/chicken/Henan/01/2004(H5N1)) hemagglutinin (HA) mRNA, complete cds.
gi|71277596|gb|DQ083550| /Avian/4(HA)/H5N1/Thailand/2004/// Influenza A virus (A/chicken/Suphanburi/Thailand/CU-1/04(H5N1)) hemagglutinin gene, partial cds.
gi|71277598|gb|DQ083551| /Avian/4(HA)/H5N1/Thailand/2004/// Influenza A virus (A/chicken/Bangkok/Thailand/CU-3/04(H5N1)) hemagglutinin gene, complete cds.
gi|71277600|gb|DQ083552| /Avian/4(HA)/H5N1/Thailand/2004/// Influenza A virus (A/crow/Bangkok/Thailand/CU-4/04(H5N1)) hemagglutinin gene, partial cds.
gi|71277602|gb|DQ083553| /Avian/4(HA)/H5N1/Thailand/2004/// Influenza A virus (A/duck/Chonburi/Thailand/CU-5/04(H5N1)) hemagglutinin gene, partial cds.
gi|71277604|gb|DQ083554| /Avian/4(HA)/H5N1/Thailand/2004/// Influenza A virus (A/chicken/Bangkok/Thailand/CU-6/04(H5N1)) hemagglutinin gene, partial cds.
gi|71277606|gb|DQ083555| /Avian/4(HA)/H5N1/Thailand/2004/// Influenza A virus (A/chicken/Chonburi/Thailand/CU-7/04(H5N1)) hemagglutinin gene, partial cds.
gi|71277608|gb|DQ083556| /Avian/4(HA)/H5N1/Thailand/2004/// Influenza A virus (A/chicken/Prachinburi/Thailand/CU-8/04(H5N1)) hemagglutinin gene, partial cds.
gi|71277610|gb|DQ083557| /Avian/4(HA)/H5N1/Thailand/2004/// Influenza A virus (A/chicken/Suphanburi/Thailand/CU-9/04(H5N1)) hemagglutinin gene, partial cds.
gi|71277612|gb|DQ083558| /Avian/4(HA)/H5N1/Thailand/2004/// Influenza A virus (A/chicken/Chachoengsao/Thailand/CU-10/04(H5N1)) hemagglutinin gene, partial cds.
gi|71277614|gb|DQ083559| /Avian/4(HA)/H5N1/Thailand/2004/// Influenza A virus (A/chicken/Chachoengsao/Thailand/CU-11/04(H5N1)) hemagglutinin gene, partial cds.
gi|71277616|gb|DQ083560| /Avian/4(HA)/H5N1/Thailand/2004/// Influenza A virus (A/chicken/Nakhon Sawan/Thailand/CU-12/04(H5N1)) hemagglutinin gene, partial cds.
gi|84797207|gb|DQ320912| /Avian/4(HA)/H5N1/China/2005/// Influenza A virus (A/duck/Hunan/1608/2005(H5N1)) hemagglutinin (HA) gene, partial cds.
gi|84797209|gb|DQ320913| /Avian/4(HA)/H5N1/China/2005/// Influenza A virus (A/duck/Hunan/1652/2005(H5N1)) hemagglutinin (HA) gene, partial cds.
gi|84797211|gb|DQ320914| /Avian/4(HA)/H5N1/China/2003/// Influenza A virus (A/duck/Shantou/4610/2003(H5N1)) hemagglutinin (HA) gene, partial cds.
gi|71277624|gb|DQ083564| /Avian/4(HA)/H5N1/Thailand/2004/// Influenza A virus (A/white peafowl/Bangkok/Thailand/CU-16/04(H5N1)) hemagglutinin gene, complete cds.
gi|71277626|gb|DQ083565| /Avian/4(HA)/H5N1/Thailand/2004/// Influenza A virus (A/chicken/Saraburi/Thailand/CU-17/04(H5N1)) hemagglutinin gene, complete cds.
gi|71277628|gb|DQ083566| /Avian/4(HA)/H5N1/Thailand/2004/// Influenza A virus (A/Kalji Pheasant/Bangkok/Thailand/CU-18/04(H5N1)) hemagglutinin gene, partial cds.
gi|71277630|gb|DQ083567| /Avian/4(HA)/H5N1/Thailand/2004/// Influenza A virus (A/Ostrich/Samut Prakan/Thailand/CU-19/04(H5N1)) hemagglutinin gene, partial cds.
gi|84797221|gb|DQ320919| /Avian/4(HA)/H5N1/China/2005/// Influenza A virus (A/migratory duck/Jiangxi/2136/2005(H5N1)) hemagglutinin (HA) gene, partial cds.
gi|84797223|gb|DQ320920| /Avian/4(HA)/H5N1/China/2005/// Influenza A virus (A/migratory duck/Jiangxi/2295/2005(H5N1)) hemagglutinin (HA) gene, partial cds.
gi|84797225|gb|DQ320921| /Avian/4(HA)/H5N1/China/2005/// Influenza A virus (A/migratory duck/Jiangxi/2300/2005(H5N1)) hemagglutinin (HA) gene, partial cds.
gi|71277638|gb|DQ083571| /Avian/4(HA)/H5N1/Thailand/2004/// Influenza A virus (A/rollers/Bangkok/Thailand/CU-26/04(H5N1)) hemagglutinin gene, partial cds.
gi|71277640|gb|DQ083572| /Avian/4(HA)/H5N1/Thailand/2004/// Influenza A virus (A/chicken/Saraburi/Thailand/CU-27/04(H5N1)) hemagglutinin gene, partial cds.
gi|71277642|gb|DQ083573| /Avian/4(HA)/H5N1/Thailand/2004/// Influenza A virus (A/white peafowl/Bangkok/Thailand/CU-29/04(H5N1)) hemagglutinin gene, complete cds.
gi|84797233|gb|DQ320925| /Avian/4(HA)/H5N1/Hong Kong/2005/// Influenza A virus (A/Chinese pond heron/Hong Kong/18/2005(H5N1)) hemagglutinin (HA) gene, partial cds.
gi|71277646|gb|DQ083575| /Avian/4(HA)/H5N1/Thailand/2004/// Influenza A virus (A/crow/Bangkok/Thailand/CU-35/04(H5N1)) hemagglutinin gene, partial cds.
gi|71277648|gb|DQ083576| /Avian/4(HA)/H5N1/Thailand/2004/// Influenza A virus (A/chicken/Lopburi/Thailand/CU-38/04(H5N1)) hemagglutinin gene, partial cds.
gi|71277650|gb|DQ083577| /Avian/4(HA)/H5N1/Thailand/2004/// Influenza A virus (A/chicken/Nakhon Sawan/Thailand/CU-39/04(H5N1)) hemagglutinin gene, partial cds.
gi|71277652|gb|DQ083578| /Avian/4(HA)/H5N1/Thailand/2004/// Influenza A virus (A/chicken/Ratchaburi/Thailand/CU-68/04(H5N1)) hemagglutinin gene, complete cds.
gi|71277654|gb|DQ083579| /Avian/4(HA)/H5N1/Thailand/2004/// Influenza A virus (A/duck/Nakhon Pathom/Thailand/CU-71/04(H5N1)) hemagglutinin gene, partial cds.
gi|71277656|gb|DQ083580| /Avian/4(HA)/H5N1/Thailand/2004/// Influenza A virus (A/chicken/Chonburi/Thailand/CU-73/04(H5N1)) hemagglutinin gene, partial cds.
gi|56119215|gb|AY720942| /Avian/4(HA)/H5N1/Viet Nam/2004/// Influenza A virus (A/chicken/Viet Nam/DT-171/2004(H5N1)) hemagglutinin (HA) gene, partial cds.
gi|56119223|gb|AY720945| /Avian/4(HA)/H5N1/Viet Nam/2004/// Influenza A virus (A/duck/Viet Nam/TG-007A/2004(H5N1)) hemagglutinin (HA) gene, partial cds.
gi|56119233|gb|AY720950| /Human/4(HA)/H5N1/Viet Nam/2004/// Influenza A virus (A/Viet Nam/DN-33/2004(H5N1)) hemagglutinin (HA) gene, partial cds.
gi|71277664|gb|DQ083584| /Avian/4(HA)/H5N1/Thailand/2004/// Influenza A virus (A/sparrow/Phang-Nga/Thailand/CU-203/04(H5N1)) hemagglutinin gene, complete cds.
gi|57924439|gb|AY724785| /Avian/4(HA)/H5N1/Viet Nam/2004/// Influenza A virus (A/chicken/Viet Nam/DN-045/2004(H5N1)) hemagglutinin (HA) gene, partial cds.
gi|57924521|gb|AY724787| /Avian/4(HA)/H5N1/Viet Nam/2004/// Influenza A virus (A/chicken/Viet Nam/VL-008/2004(H5N1)) hemagglutinin (HA) gene, partial cds.
gi|91984119|gb|DQ095613| /Avian/4(HA)/H5N1/China/2005/// Influenza A virus (A/Bar-headed Goose/Qinghai/68/05(H5N1)) hemagglutinin (HA) gene, partial cds.
gi|70955423|gb|DQ095614| /Avian/4(HA)/H5N1/China/2005/// Influenza A virus (A/Great Black-headed Gull/Qinghai/2/05(H5N1)) hemagglutinin (HA) gene, partial cds.
gi|70955425|gb|DQ095615| /Avian/4(HA)/H5N1/China/2005/// Influenza A virus (A/Bar-headed Goose/Qinghai/60/05(H5N1)) hemagglutinin (HA) gene, partial cds.
gi|91984122|gb|DQ095616| /Avian/4(HA)/H5N1/China/2005/// Influenza A virus (A/Brown-headed Gull/Qinghai/3/05(H5N1)) hemagglutinin (HA) gene, partial cds.
gi|85062766|gb|DQ334760| /Avian/4(HA)/H5N1/Thailand/2005/// Influenza A virus (A/chicken/Thailand/Kanchanaburi/CK-160/2005(H5N1)) hemagglutinin gene, complete cds.
gi|51628336|gb|AY728894| /Avian/4(HA)/H5N1/Viet Nam/2004/// Influenza A virus (A/chicken/Viet Nam/HauGiang-617/2004(H5N1)) hemagglutinin (HA) gene, partial cds.
gi|57915979|gb|AY737289| /Avian/4(HA)/H5N1/China/2004/// Influenza A virus (A/chicken/Guangdong/191/04(H5N1)) segment 4, complete sequence.
gi|70955431|gb|DQ095620| /Avian/4(HA)/H5N1/China/2005/// Influenza A virus (A/Bar-headed Goose/Qinghai/62/05(H5N1)) hemagglutinin (HA) gene, partial cds.
gi|91984128|gb|DQ095621| /Avian/4(HA)/H5N1/China/2005/// Influenza A virus (A/Bar-headed Goose/Qinghai/12/05(H5N1)) hemagglutinin (HA) gene, partial cds.
gi|56672736|gb|AY741213| /Avian/4(HA)/H5N1/China/2004/// Influenza A virus (A/blackbird/Hunan/1/2004(H5N1)) hemagglutinin (HA) gene, complete cds.
gi|56672789|gb|AY741215| /Avian/4(HA)/H5N1/China/2004/// Influenza A virus (A/tree sparrow/Henan/1/2004(H5N1)) hemagglutinin (HA) gene, complete cds.
gi|56672873|gb|AY741217| /Avian/4(HA)/H5N1/China/2004/// Influenza A virus (A/tree sparrow/Henan/2/2004(H5N1)) hemagglutinin (HA) gene, complete cds.
gi|56672930|gb|AY741219| /Avian/4(HA)/H5N1/China/2004/// Influenza A virus (A/tree sparrow/Henan/3/2004(H5N1)) hemagglutinin (HA) gene, complete cds.
gi|56673032|gb|AY741221| /Avian/4(HA)/H5N1/China/2004/// Influenza A virus (A/tree sparrow/Henan/4/2004(H5N1)) hemagglutinin (HA) gene, complete cds.

gi|54126496|gb|AY747609| /Swine/4(HA)/H5N1/China/2003/// Influenza A virus (A/swine/Fujian/1/2003(H5N1)) hemagglutinin (HA) gene, complete cds.
gi|54126527|gb|AY747617| /Swine/4(HA)/H5N1/China/2001/// Influenza A virus (A/swine/Fujian/F1/2001(H5N1)) hemagglutinin (HA) gene, complete cds.
gi|55233227|gb|AY770079| /Avian/4(HA)/H5N1/China/2004/// Influenza A virus (A/chicken/Hubei/489/2004(H5N1)) hemagglutinin (HA) gene, complete cds.
gi|54873457|gb|AY770991| /Avian/4(HA)/H5N1/Thailand/2004/// Influenza A virus (A/chicken/Ayutthaya/Thailand/CU-23/04(H5N1)) hemagglutinin gene, partial cds.
gi|70955447|gb|DQ095631| /Avian/4(HA)/H5N1/China/2005/// Influenza A virus (A/Duck/Hunan/191/05(H5N1)) hemagglutinin (HA) gene, partial cds.
gi|55247881|gb|AY779050| /Avian/4(HA)/H5N1/Thailand/2004/// Influenza A virus (A/chicken/Thailand/CU-21/2004 (H5N1)) hemagglutinin gene, partial cds.
gi|72398642|gb|DQ099756| /Avian/4(HA)/H5N1/Viet Nam/2004/// Influenza A virus (A/duck/Viet Nam/TG-007A/2004(H5N1)) segment 4 hemagglutinin (HA) gene, complete cds.
gi|58618439|gb|AY818136| /Avian/4(HA)/H5N1/Viet Nam/2004/// Influenza A virus (A/chicken/Vietnam/C58/04(H5N1)) hemagglutinin HA gene, complete cds.
gi|58618441|gb|AY818137| /Avian/4(HA)/H5N1/Viet Nam/2004/// Influenza A virus (A/quail/Vietnam/36/04(H5N1)) hemagglutinin HA gene, complete cds.
gi|56418513|gb|AY830774| /Avian/4(HA)/H5N1/China/2004/// Influenza A virus (A/chick/Macheng/2004(H5N1)) hemagglutinin (HA) gene, complete cds.
gi|56553497|gb|AY834279| /Tiger/4(HA)/H5N1/Thailand/2004/// Influenza A virus (A/tiger/Thailand/SPB-1(H5N1)) hemagglutinin gene, complete cds.
gi|56792949|gb|AY842935| /Tiger/4(HA)/H5N1/Thailand/2004/// Influenza A virus (A/tiger/Thailand/CU-T3/2004(H5N1)) hemagglutinin gene, partial cds.
gi|58374179|gb|AY854190| /Avian/4(HA)/H5N1/China/2004/// Influenza A virus (A/duck/Shandong/093/2004(H5N1)) segment 4, complete sequence.
gi|71068504|gb|DQ100557| /Avian/4(HA)/H5N1/China/2005/// Influenza A virus (A/great black-headed gull/Qinghai/1/2005(H5N1)) hemagglutinin gene, partial cds; and HA gene, partial sequence.
gi|58198724|gb|AY866475| /Tiger/4(HA)/H5N1/Thailand/2004/// Influenza A virus (A/tiger/Thailand/CU-T7/2004(H5N1)) hemagglutinin gene, complete cds.
gi|61698013|gb|AY950230| /Avian/4(HA)/H5N1/China/2004/// Influenza A virus (A/chicken/Henan/01/2004(H5N1)) segment 4, complete sequence.
gi|61698015|gb|AY950231| /Avian/4(HA)/H5N1/China/2004/// Influenza A virus (A/Chicken/Henan/210/2004 (H5N1)) segment 4, complete sequence.
gi|61698017|gb|AY950232| /Avian/4(HA)/H5N1/China/2004/// Influenza A virus (A/Chicken/Henan/12/2004 (H5N1)) segment 4, complete sequence.
gi|61698019|gb|AY950233| /Avian/4(HA)/H5N1/China/2004/// Influenza A virus (A/Chicken/Henan/13/2004 (H5N1)) segment 4, complete sequence.
gi|61698021|gb|AY950234| /Avian/4(HA)/H5N1/China/2004/// Influenza A virus (A/Chicken/Henan/16/2004 (H5N1)) segment 4, complete sequence.
gi|61698023|gb|AY950235| /Avian/4(HA)/H5N1/China/2004/// Influenza A virus (A/WildDuck/Guangdong/314/2004 (H5N1)) segment 4, complete sequence.
gi|61698025|gb|AY950236| /Avian/4(HA)/H5N1/China/2004/// Influenza A virus (A/swan/Guangxi/307/2004 (H5N1)) segment 4, complete sequence.
gi|62466138|gb|AY972539| /Tiger/4(HA)/H5N1/Thailand/2004/// Influenza A virus (A/tiger/Thailand/CU-T4/04(H5N1)) hemagglutinin (HA) gene, complete cds.
gi|76786306|gb|DQ201829| /Avian/4(HA)/H5N1/China/2000/// Influenza A virus (A/Goose/Huadong/1/2000(H5N1)) hemagglutinin (HA) mRNA, complete cds.
gi|76800613|gb|DQ211922| /Avian/4(HA)/H5N1/China/2003/// Influenza A virus (A/chicken/jiyuan/1/03/(H5N1)) hemagglutinin (HA) gene, complete cds.
gi|76800615|gb|DQ211923| /Avian/4(HA)/H5N1/China/2002/// Influenza A virus (A/chicken/zhengzhou/1/02/(H5N1)) hemagglutinin (HA) gene, complete cds.
gi|76800617|gb|DQ211924| /Avian/4(HA)/H5N1/China/2002/// Influenza A virus (A/chicken/zhoukou/2/02/(H5N1)) hemagglutinin (HA) gene, complete cds.
gi|110613481|gb|DQ840533| /Avian/4(HA)/H5N1/Russia/2005/// Influenza A virus (A/swan/Astrakhan/Russia/Nov-2/2005(H5N1)) hemagglutinin (HA) gene, partial cds.
gi|90654261|gb|DQ458992| /Avian/4(HA)/H5N1/Germany/2006/// Influenza A virus (A/mallard/Bavaria/1/2006(H5N1)) hemagglutinin (HA) gene, complete cds.
gi|78450309|gb|DQ230521| /Avian/4(HA)/H5N1/Russia/2005/// Influenza A virus (A/grebe/Novosibirsk/29/2005(H5N1)) hemagglutinin (HA) gene, complete cds.
gi|78450348|gb|DQ230522| /Avian/4(HA)/H5N1/Russia/2005/// Influenza A virus (A/duck/Novosibirsk/56/2005(H5N1)) hemagglutinin (HA) gene, complete cds.
gi|78146041|gb|DQ231240| /Avian/4(HA)/H5N1/Russia/2005/// Influenza A virus (A/turkey/Suzdalka/Nov-01/05(H5N1)) hemagglutinin (HA) gene, partial cds.
gi|93008236|gb|DQ497644| /Avian/4(HA)/H5N1/Indonesia/2004/// Influenza A virus (A/chicken/Ngawi/BPPV4/2004(H5N1)) hemagglutinin (HA) gene, partial cds.
gi|93008318|gb|DQ497685| /Avian/4(HA)/H5N1/Viet Nam/2004/// Influenza A virus (A/duck/Vietnam/220/2004(H5N1)) hemagglutinin (HA) gene, partial cds.
gi|78458570|gb|DQ236077| /Cat/4(HA)/H5N1/Thailand/2004/// Influenza A virus (A/cat/Thailand/KU-02/04(H5N1)) hemagglutinin (HA) gene, complete cds.
gi|78458879|gb|DQ236085| /Avian/4(HA)/H5N1/Thailand/2004/// Influenza A virus (A/pigeon/Thailand/KU-03/04(H5N1)) hemagglutinin (HA) gene, complete cds.
gi|93008244|gb|DQ497648| /Avian/4(HA)/H5N1/Indonesia/2005/// Influenza A virus (A/chicken/Purworejo/BBVW/2005(H5N1)) hemagglutinin (HA) gene, partial cds.
gi|93008246|gb|DQ497649| /Avian/4(HA)/H5N1/Indonesia/2004/// Influenza A virus (A/quail/Yogjakarta/BBVet-IX/2004(H5N1)) hemagglutinin (HA) gene, partial cds.
gi|93008248|gb|DQ497650| /Avian/4(HA)/H5N1/Indonesia/2004/// Influenza A virus (A/chicken/Kulon Progo/BBVet-XII-2/2004(H5N1)) hemagglutinin (HA) gene, partial cds.
gi|93008250|gb|DQ497651| /Avian/4(HA)/H5N1/Indonesia/2005/// Influenza A virus (A/chicken/Gunung Kidal/BBVW/2005(H5N1)) hemagglutinin (HA) gene, partial cds.
gi|93008252|gb|DQ497652| /Avian/4(HA)/H5N1/Indonesia/2005/// Influenza A virus (A/chicken/Kulon Progo/BBVW/2005(H5N1)) hemagglutinin (HA) gene, partial cds.
gi|82621197|gb|DQ279301| /Avian/4(HA)/H5N1/Russia/2005/// Influenza A virus (A/chicken/Tula/10/2005(H5N1)) hemagglutinin (HA) gene, partial cds.
gi|84627165|gb|DQ320137| /Avian/4(HA)/H5N1/Russia/2005/// Influenza A virus (A/swan/Astrakhan/1/2005(H5N1)) hemagglutinin (HA) gene, partial cds.
gi|84797133|gb|DQ320875| /Avian/4(HA)/H5N1/China/2005/// Influenza A virus (A/duck/Fujian/897/2005(H5N1)) hemagglutinin (HA) gene, partial cds.
gi|84797135|gb|DQ320876| /Avian/4(HA)/H5N1/China/2005/// Influenza A virus (A/chicken/Fujian/1042/2005(H5N1)) hemagglutinin (HA) gene, partial cds.
gi|84797137|gb|DQ320877| /Avian/4(HA)/H5N1/China/2004/// Influenza A virus (A/duck/Guangxi/351/2004(H5N1)) hemagglutinin (HA) gene, partial cds.
gi|84797139|gb|DQ320878| /Avian/4(HA)/H5N1/China/2004/// Influenza A virus (A/duck/Guangxi/380/2004(H5N1)) hemagglutinin (HA) gene, partial cds.
gi|84797141|gb|DQ320879| /Avian/4(HA)/H5N1/China/2004/// Influenza A virus (A/duck/Guangxi/668/2004(H5N1)) hemagglutinin (HA) gene, partial cds.
gi|84797143|gb|DQ320880| /Avian/4(HA)/H5N1/China/2004/// Influenza A virus (A/goose/Guangxi/914/2004(H5N1)) hemagglutinin (HA) gene, partial cds.
gi|84797145|gb|DQ320881| /Avian/4(HA)/H5N1/China/2004/// Influenza A virus (A/goose/Guangxi/1097/2004(H5N1)) hemagglutinin (HA) gene, partial cds.
gi|84797147|gb|DQ320882| /Avian/4(HA)/H5N1/China/2004/// Influenza A virus (A/goose/Guangxi/1198/2004(H5N1)) hemagglutinin (HA) gene, partial cds.
gi|84797149|gb|DQ320883| /Avian/4(HA)/H5N1/China/2004/// Influenza A virus (A/duck/Guangxi/1311/2004(H5N1)) hemagglutinin (HA) gene, partial cds.
gi|93008276|gb|DQ497664| /Avian/4(HA)/H5N1/Indonesia/2004/// Influenza A virus (A/turkey/Kedaton/BPPV3/2004(H5N1)) hemagglutinin (HA) gene, partial cds.
gi|84797153|gb|DQ320885| /Avian/4(HA)/H5N1/China/2004/// Influenza A virus (A/duck/Guangxi/1586/2004(H5N1)) hemagglutinin (HA) gene, partial cds.
gi|84797155|gb|DQ320886| /Avian/4(HA)/H5N1/China/2004/// Influenza A virus (A/duck/Guangxi/1681/2004(H5N1)) hemagglutinin (HA) gene, partial cds.
gi|84797157|gb|DQ320887| /Avian/4(HA)/H5N1/China/2004/// Influenza A virus (A/duck/Guangxi/1793/2004(H5N1)) hemagglutinin (HA) gene, partial cds.
gi|84797159|gb|DQ320888| /Avian/4(HA)/H5N1/China/2004/// Influenza A virus (A/goose/Guangxi/1832/2004(H5N1)) hemagglutinin (HA) gene, partial cds.
gi|84797161|gb|DQ320889| /Avian/4(HA)/H5N1/China/2004/// Influenza A virus (A/goose/Guangxi/2112/2004(H5N1)) hemagglutinin (HA) gene, partial cds.
gi|84797163|gb|DQ320890| /Avian/4(HA)/H5N1/China/2004/// Influenza A virus (A/duck/Guangxi/2291/2004(H5N1)) hemagglutinin (HA) gene, partial cds.
gi|84797165|gb|DQ320891| /Avian/4(HA)/H5N1/China/2004/// Influenza A virus (A/goose/Guangxi/2383/2004(H5N1)) hemagglutinin (HA) gene, partial cds.
gi|84797167|gb|DQ320892| /Avian/4(HA)/H5N1/China/2004/// Influenza A virus (A/duck/Guangxi/2396/2004(H5N1)) hemagglutinin (HA) gene, partial cds.
gi|84797169|gb|DQ320893| /Avian/4(HA)/H5N1/China/2004/// Influenza A virus (A/chicken/Guangxi/2439/2004(H5N1)) hemagglutinin (HA) gene, partial cds.
gi|84797171|gb|DQ320894| /Avian/4(HA)/H5N1/China/2004/// Influenza A virus (A/chicken/Guangxi/2448/2004(H5N1)) hemagglutinin (HA) gene, partial cds.
gi|93008298|gb|DQ497675| /Avian/4(HA)/H5N1/Viet Nam/2005/// Influenza A virus (A/mallard/Vietnam/347/2005(H5N1)) hemagglutinin (HA) gene, partial cds.
gi|93008300|gb|DQ497676| /Avian/4(HA)/H5N1/Viet Nam/2005/// Influenza A virus (A/chicken/Vietnam/348/2005(H5N1)) hemagglutinin (HA) gene, partial cds.
gi|93008302|gb|DQ497677| /Avian/4(HA)/H5N1/Viet Nam/2005/// Influenza A virus (A/mallard/Vietnam/352/2005(H5N1)) hemagglutinin (HA) gene, partial cds.
gi|93008304|gb|DQ497678| /Avian/4(HA)/H5N1/Viet Nam/2003/// Influenza A virus (A/chicken/Vietnam/19/2003(H5N1)) hemagglutinin (HA) gene, partial cds.
gi|93008306|gb|DQ497679| /Avian/4(HA)/H5N1/Viet Nam/2003/// Influenza A virus (A/chicken/Vietnam/20/2003(H5N1)) hemagglutinin (HA) gene, partial cds.
gi|84797183|gb|DQ320900| /Avian/4(HA)/H5N1/China/2005/// Influenza A virus (A/duck/Guangxi/951/2005(H5N1)) hemagglutinin (HA) gene, partial cds.
gi|84797185|gb|DQ320901| /Avian/4(HA)/H5N1/China/2005/// Influenza A virus (A/duck/Guangzhou/20/2005(H5N1)) hemagglutinin (HA) gene, partial cds.
gi|84797187|gb|DQ320902| /Avian/4(HA)/H5N1/China/2005/// Influenza A virus (A/duck/Hunan/127/2005(H5N1)) hemagglutinin (HA) gene, partial cds.
gi|84797189|gb|DQ320903| /Avian/4(HA)/H5N1/China/2005/// Influenza A virus (A/duck/Hunan/139/2005(H5N1)) hemagglutinin (HA) gene, partial cds.
gi|84797191|gb|DQ320904| /Avian/4(HA)/H5N1/China/2005/// Influenza A virus (A/duck/Hunan/149/2005(H5N1)) hemagglutinin (HA) gene, partial cds.
gi|84797193|gb|DQ320905| /Avian/4(HA)/H5N1/China/2005/// Influenza A virus (A/duck/Hunan/152/2005(H5N1)) hemagglutinin (HA) gene, partial cds.
gi|84797195|gb|DQ320906| /Avian/4(HA)/H5N1/China/2005/// Influenza A virus (A/duck/Hunan/157/2005(H5N1)) hemagglutinin (HA) gene, partial cds.
gi|84797197|gb|DQ320907| /Avian/4(HA)/H5N1/China/2005/// Influenza A virus (A/duck/Hunan/160/2005(H5N1)) hemagglutinin (HA) gene, partial cds.
gi|84797199|gb|DQ320908| /Avian/4(HA)/H5N1/China/2005/// Influenza A virus (A/duck/Hunan/166/2005(H5N1)) hemagglutinin (HA) gene, partial cds.
gi|84797201|gb|DQ320909| /Avian/4(HA)/H5N1/China/2005/// Influenza A virus (A/duck/Hunan/182/2005(H5N1)) hemagglutinin (HA) gene, partial cds.
gi|84797203|gb|DQ320910| /Avian/4(HA)/H5N1/China/2005/// Influenza A virus (A/chicken/Hunan/999/2005(H5N1)) hemagglutinin (HA) gene, partial cds.
gi|84797205|gb|DQ320911| /Avian/4(HA)/H5N1/China/2005/// Influenza A virus (A/duck/Hunan/1265/2005(H5N1)) hemagglutinin (HA) gene, partial cds.
gi|93008332|gb|DQ497692| /Avian/4(HA)/H5N1/Viet Nam/2003/// Influenza A virus (A/chicken/Vietnam/5/2003(H5N1)) hemagglutinin (HA) gene, partial cds.
gi|93008334|gb|DQ497693| /Avian/4(HA)/H5N1/Viet Nam/2003/// Influenza A virus (A/chicken/Vietnam/8/2003(H5N1)) hemagglutinin (HA) gene, partial cds.
gi|93008336|gb|DQ497694| /Avian/4(HA)/H5N1/Viet Nam/2005/// Influenza A virus (A/duck/Vietnam/S640/2005(H5N1)) hemagglutinin (HA) gene, partial cds.
gi|84797213|gb|DQ320915| /Avian/4(HA)/H5N1/China/2005/// Influenza A virus (A/goose/Shantou/2216/2005(H5N1)) hemagglutinin (HA) gene, partial cds.
gi|84797215|gb|DQ320916| /Avian/4(HA)/H5N1/China/2005/// Influenza A virus (A/migratory duck/Jiangxi/1653/2005(H5N1)) hemagglutinin (HA) gene, partial cds.
gi|84797217|gb|DQ320917| /Avian/4(HA)/H5N1/China/2005/// Influenza A virus (A/migratory duck/Jiangxi/1657/2005(H5N1)) hemagglutinin (HA) gene, partial cds.
gi|84797219|gb|DQ320918| /Avian/4(HA)/H5N1/China/2005/// Influenza A virus (A/migratory duck/Jiangxi/1701/2005(H5N1)) hemagglutinin (HA) gene, partial cds.
gi|86753761|gb|DQ366330| /Avian/4(HA)/H5N1/China/2004/// Influenza A virus (A/chicken/Guangxi/12/2004(H5N1)) hemagglutinin mRNA, complete cds.
gi|93008348|gb|DQ497700| /Avian/4(HA)/H5N1/Viet Nam/2004/// Influenza A virus (A/chicken/Vietnam/133/2004(H5N1)) hemagglutinin (HA) gene, partial cds.
gi|93008350|gb|DQ497701| /Avian/4(HA)/H5N1/Viet Nam/2004/// Influenza A virus (A/chicken/Vietnam/147/2004(H5N1)) hemagglutinin (HA) gene, partial cds.
gi|84797227|gb|DQ320922| /Environment/4(HA)/H5N1/China/2005/// Influenza A virus (A/Environment/Qinghai/31/2005(H5N1)) hemagglutinin (HA) gene, partial cds.
gi|84797229|gb|DQ320923| /Avian/4(HA)/H5N1/Hong Kong/2004/// Influenza A virus (A/grey heron/Hong Kong/728/2004(H5N1)) hemagglutinin (HA) gene, partial cds.
gi|84797231|gb|DQ320924| /Avian/4(HA)/H5N1/Hong Kong/2004/// Influenza A virus (A/grey heron/Hong Kong/837/2004(H5N1)) hemagglutinin (HA) gene, partial cds.

gi|93008358|gb|DQ497705| /Avian/4(HA)/H5N1/Viet Nam/2005/// Influenza A virus (A/wild bird/Vietnam/434/2005(H5N1)) hemagglutinin (HA) gene, partial cds.
gi|84797235|gb|DQ320926| /Avian/4(HA)/H5N1/Hong Kong/2002/// Influenza A virus (A/chicken/Hong Kong/3123.1/2002(H5N1)) hemagglutinin (HA) gene, partial cds.
gi|84797237|gb|DQ320927| /Avian/4(HA)/H5N1/Hong Kong/2002/// Influenza A virus (A/chicken/Hong Kongi/86.3/2002(H5N1)) hemagglutinin (HA) gene, partial cds.
gi|84797239|gb|DQ320928| /Avian/4(HA)/H5N1/Indonesia/2005/// Influenza A virus (A/chicken/Salatiga/BBVet-I/2005(H5N1)) hemagglutinin (HA) gene, partial cds.
gi|84797241|gb|DQ320929| /Avian/4(HA)/H5N1/Indonesia/2003/// Influenza A virus (A/chicken/Wonosobo/BPPV4/2003(H5N1)) hemagglutinin (HA) gene, partial cds.
gi|84797243|gb|DQ320930| /Avian/4(HA)/H5N1/Indonesia/2004/// Influenza A virus (A/chicken/Yogjakarta/BBVet-IX/2004(H5N1)) hemagglutinin (HA) gene, partial cds.
gi|84797245|gb|DQ320931| /Avian/4(HA)/H5N1/Indonesia/2004/// Influenza A virus (A/chicken/Kulon Progo/BBVet-XII-1/2004(H5N1)) hemagglutinin (HA) gene, partial cds.
gi|71277658|gb|DQ083581| /Avian/4(HA)/H5N1/Thailand/2004/// Influenza A virus (A/duck/Saraburi/Thailand/CU-74/04(H5N1)) hemagglutinin gene, partial cds.
gi|71277660|gb|DQ083582| /Avian/4(HA)/H5N1/Thailand/2004/// Influenza A virus (A/chicken/Prachinburi/Thailand/CU-104/04(H5N1)) hemagglutinin gene, partial cds.
gi|71277662|gb|DQ083583| /Avian/4(HA)/H5N1/Thailand/2004/// Influenza A virus (A/pigeon/Samut Prakan/Thailand/CU-202/04(H5N1)) hemagglutinin gene, complete cds.
gi|84797253|gb|DQ320935| /Avian/4(HA)/H5N1/Malaysia/2004/// Influenza A virus (A/quail/Malaysia/6309/2004(H5N1)) hemagglutinin (HA) gene, partial cds.
gi|71277666|gb|DQ083585| /Avian/4(HA)/H5N1/Thailand/2004/// Influenza A virus (A/Mynas/Ranong/Thailand/CU-209/04(H5N1)) hemagglutinin gene, complete cds.
gi|91984117|gb|DQ095612| /Avian/4(HA)/H5N1/China/2005/// Influenza A virus (A/Bar-headed Goose/Qinghai/59/05(H5N1)) hemagglutinin (HA) gene, partial cds.
gi|84797259|gb|DQ320938| /Avian/4(HA)/H5N1/Viet Nam/2003/// Influenza A virus (A/chicken/Vietnam/27/2003(H5N1)) hemagglutinin (HA) gene, partial cds.
gi|84797261|gb|DQ320939| /Avian/4(HA)/H5N1/Viet Nam/2005/// Influenza A virus (A/duck/Vietnam/568/2005(H5N1)) hemagglutinin (HA) gene, partial cds.
gi|84797263|gb|DQ320940| /Avian/4(HA)/H5N1/Viet Nam/2004/// Influenza A virus (A/Mallard duck/Vietnam/133/2004(H5N1)) hemagglutinin (HA) gene, partial cds.
gi|83940767|gb|DQ323672| /Avian/4(HA)/H5N1/Russia/2005/// Influenza A virus (A/chicken/Kurgan/3/2005(H5N1)) hemagglutinin precursor (HA) gene, complete cds.
gi|93008392|gb|DQ497722| /Human/4(HA)/H5N1/Viet Nam/2004/// Influenza A virus (A/Vietnam/CL20/2004(H5N1)) hemagglutinin (HA) gene, partial cds.
gi|91984126|gb|DQ095618| /Avian/4(HA)/H5N1/China/2005/// Influenza A virus (A/Bar-headed Goose/Qinghai/61/05(H5N1)) hemagglutinin (HA) gene, partial cds.
gi|70955429|gb|DQ095619| /Avian/4(HA)/H5N1/China/2005/// Influenza A virus (A/Bar-headed Goose/Qinghai/75/05(H5N1)) hemagglutinin (HA) gene, partial cds.
gi|85372720|gb|DQ340848| /Avian/4(HA)/H5N1/Ukraine/2005/// Influenza A virus (A/chicken/Crimea/1/2005(H5N1)) hemagglutinin (HA) gene, partial cds.
gi|93008400|gb|DQ497726| /Human/4(HA)/H5N1/Viet Nam/2005/// Influenza A virus (A/Vietnam/CL105/2005(H5N1)) hemagglutinin (HA) gene, partial cds.
gi|70955434|gb|DQ095622| /Avian/4(HA)/H5N1/China/2005/// Influenza A virus (A/Bar-headed Goose/Qinghai/65/05(H5N1)) hemagglutinin (HA) gene, partial cds.
gi|70955436|gb|DQ095623| /Avian/4(HA)/H5N1/China/2005/// Influenza A virus (A/Bar-headed Goose/Qinghai/67/05(H5N1)) hemagglutinin (HA) gene, partial cds.
gi|91984130|gb|DQ095624| /Avian/4(HA)/H5N1/China/2005/// Influenza A virus (A/Chicken/Yunnan/447/05(H5N1)) hemagglutinin (HA) gene, partial cds.
gi|91984132|gb|DQ095625| /Avian/4(HA)/H5N1/China/2005/// Influenza A virus (A/Chicken/Yunnan/493/05(H5N1)) hemagglutinin (HA) gene, partial cds.
gi|70955440|gb|DQ095626| /Avian/4(HA)/H5N1/China/2005/// Influenza A virus (A/Chicken/Shantou/810/05(H5N1)) hemagglutinin (HA) gene, partial cds.
gi|87204335|gb|DQ363918| /Avian/4(HA)/H5N1/Russia/2005/// Influenza A virus (A/Cygnus olor/Astrakhan/Ast05-2-4/2005(H5N1)) hemagglutinin (HA) gene, complete cds.
gi|70955443|gb|DQ095628| /Avian/4(HA)/H5N1/China/2005/// Influenza A virus (A/Goose/Shantou/1621/05(H5N1)) hemagglutinin (HA) gene, partial cds.
gi|91984137|gb|DQ095629| /Avian/4(HA)/H5N1/China/2005/// Influenza A virus (A/Duck/Fujian/1734/05(H5N1)) hemagglutinin (HA) gene, partial cds.
gi|70955445|gb|DQ095630| /Avian/4(HA)/H5N1/China/2005/// Influenza A virus (A/Duck/Hunan/114/05(H5N1)) hemagglutinin (HA) gene, partial cds.
gi|86753755|gb|DQ366306| /Avian/4(HA)/H5N1/Viet Nam/2005/// Influenza A virus (A/duck/Vietnam/1/2005(H5N1)) hemagglutinin mRNA, complete cds.
gi|72398640|gb|DQ099755| /Avian/4(HA)/H5N1/Viet Nam/2004/// Influenza A virus (A/chicken/Viet Nam/TN-025/2004(H5N1)) segment 4 hemagglutinin (HA) gene, complete cds.
gi|86753759|gb|DQ366322| /Avian/4(HA)/H5N1/Viet Nam/2005/// Influenza A virus (A/duck/Vietnam/8/05(H5N1)) hemagglutinin mRNA, complete cds.
gi|72398644|gb|DQ099757| /Avian/4(HA)/H5N1/Viet Nam/2004/// Influenza A virus (A/quail/Viet Nam/TG-007B/2004(H5N1)) segment 4 hemagglutinin (HA) gene, complete cds.
gi|72398646|gb|DQ099758| /Avian/4(HA)/H5N1/Viet Nam/2004/// Influenza A virus (A/chicken/Viet Nam/TG-023/2004(H5N1)) segment 4 hemagglutinin (HA) gene, complete cds.
gi|72398648|gb|DQ099759| /Avian/4(HA)/H5N1/Viet Nam/2004/// Influenza A virus (A/chicken/Viet Nam/DT-171/2004(H5N1)) segment 4 hemagglutinin (HA) gene, complete cds.
gi|72398650|gb|DQ099760| /Avian/4(HA)/H5N1/Viet Nam/2004/// Influenza A virus (A/chicken/Viet Nam/LD-080/2004(H5N1)) segment 4 hemagglutinin (HA) gene, complete cds.
gi|71025271|gb|DQ100554| /Avian/4(HA)/H5N1/China/2005/// Influenza A virus (A/black-headed goose/Qinghai/1/2005(H5N1)) hemagglutinin (HA) gene, partial cds.
gi|71025273|gb|DQ100555| /Avian/4(HA)/H5N1/China/2005/// Influenza A virus (A/black-headed goose/Qinghai/2/2005(H5N1)) hemagglutinin (HA) gene, partial cds.
gi|71025275|gb|DQ100556| /Avian/4(HA)/H5N1/China/2005/// Influenza A virus (A/black-headed gull/Qinghai/1/2005(H5N1)) hemagglutinin (HA) gene, partial cds.
gi|89077643|gb|DQ399540| /Avian/4(HA)/H5N1/Russia/2005/11// Influenza A virus (A/Cygnus olor/Astrakhan/Ast05-2-8/2005(H5N1)) hemagglutinin (HA) gene, complete cds.
gi|75911269|gb|DQ137873| /Avian/4(HA)/H5N1/China/2005/// Influenza A virus (A/bar-headed goose/Qinghai/0510/05(H5N1)) hemagglutinin (HA) gene, partial cds.
gi|88604738|gb|DQ406728| /Avian/4(HA)/H5N1/Nigeria/2006/01/17/ Influenza A virus (A/chicken/Nigeria/641/2006(H5N1)) hemagglutinin (HA) gene, partial cds.
gi|89475499|gb|DQ407519| /Avian/4(HA)/H5N1/Turkey/2005/// Influenza A virus (A/turkey/Turkey/1/2005(H5N1)) hemagglutinin (HA) gene, complete cds.
gi|77378033|gb|DQ182483| /Avian/4(HA)/H5N1/Belgium/2004/// Influenza A virus (A/crested eagle/Belgium/01/2004(H5N1)) hemagglutinin (HA) gene, complete cds.
gi|89258382|gb|DQ434889| /Avian/4(HA)/H5N1/Russia/2005/11/26/ Influenza A virus (A/Cygnus olor/Astrakhan/Ast05-2-10/2005(H5N1)) hemagglutinin (HA) gene, complete cds.
gi|89275862|gb|DQ435200| /Cat/4(HA)/H5N1/Iraq/2006/// Influenza A virus (A/domestic cat/Iraq/820/2006(H5N1)) hemagglutinin (HA) gene, partial cds.
gi|89275864|gb|DQ435201| /Avian/4(HA)/H5N1/Iraq/2006/// Influenza A virus (A/domestic goose/Iraq/812/2006(H5N1)) hemagglutinin (HA) gene, partial cds.
gi|89275866|gb|DQ435202| /Human/4(HA)/H5N1/Iraq/2006/// Influenza A virus (A/human/Iraq/207-NAMRU3/2006(H5N1)) hemagglutinin (HA) gene, partial cds.
gi|89477112|gb|DQ440535| /Avian/4(HA)/H5N1/Iran/2006/02// Influenza A virus (A/Cygnus cygnus/Iran/754/2006(H5N1)) hemagglutinin (HA) gene, partial cds.
gi|90025360|gb|DQ447199| /Avian/4(HA)/H5N1/Egypt/2006/// Influenza A virus (A/chicken/Egypt/960N3-004/2006(H5N1)) hemagglutinin (HA) gene, partial cds.
gi|89994020|gb|DQ449031| /Avian/4(HA)/H5N1/Italy/2006/02// Influenza A virus (A/mallard/Italy/835/2006(H5N1)) hemagglutinin (HA) gene, partial cds.
gi|90289625|gb|DQ449632| /Avian/4(HA)/H5N1/Russia/2005/// Influenza A virus (A/chicken/Kurgan/05/2005(H5N1)) hemagglutinin (HA) mRNA, complete cds.
gi|90289674|gb|DQ449640| /Avian/4(HA)/H5N1/Russia/2005/// Influenza A virus (A/duck/Kurgan/08/2005(H5N1)) hemagglutinin (HA) mRNA, complete cds.
gi|21326684|gb|AY075033| /Avian/4(HA)/H5N1/Hong Kong/2001/// Influenza A virus (A/Duck/Hong Kong/380.5/2001(H5N1)) hemagglutinin H5 (H5) gene, complete cds.
gi|110555220|gb|DQ852600| /Avian/4(HA)/H5N1/Russia/2006/06/24/ Influenza A virus (A/grebe/Tyva/Tyv06-2/06(H5N1)) hemagglutinin (HA) gene, complete cds.
gi|90823033|gb|DQ464377| /Human/4(HA)/H5N1/Egypt/2006/// Influenza A virus (A/Egypt/2782-NAMRU3/2006(H5N1)) hemagglutinin (HA) gene, partial cds.
gi|93008232|gb|DQ497642| /Avian/4(HA)/H5N1/Indonesia/2004/// Influenza A virus (A/chicken/Malang/BBVet-IV/2004(H5N1)) hemagglutinin (HA) gene, partial cds.
gi|93008234|gb|DQ497643| /Avian/4(HA)/H5N1/Indonesia/2005/// Influenza A virus (A/chicken/Magetan/BBVW/2005(H5N1)) hemagglutinin (HA) gene, partial cds.
gi|93008316|gb|DQ497684| /Avian/4(HA)/H5N1/Viet Nam/2004/// Influenza A virus (A/duck/Vietnam/219/2004(H5N1)) hemagglutinin (HA) gene, partial cds.
gi|110631388|gb|DQ862002| /Avian/4(HA)/H5N1/Egypt/2006/06// Influenza A virus (A/duck/Egypt/2253-3/2006(H5N1)) hemagglutinin (HA) gene, complete cds.
gi|93008240|gb|DQ497646| /Avian/4(HA)/H5N1/Indonesia/2003/// Influenza A virus (A/chicken/Sragen/BPPV4/2003(H5N1)) hemagglutinin (HA) gene, partial cds.
gi|93008242|gb|DQ497647| /Avian/4(HA)/H5N1/Indonesia/2004/// Influenza A virus (A/quail/Boyolali/BPPV4/2004(H5N1)) hemagglutinin (HA) gene, partial cds.
gi|93008324|gb|DQ497688| /Avian/4(HA)/H5N1/Viet Nam/2005/// Influenza A virus (A/duck/Vietnam/N-TB/2005(H5N1)) hemagglutinin (HA) gene, partial cds.
gi|93008326|gb|DQ497689| /Avian/4(HA)/H5N1/Viet Nam/2005/// Influenza A virus (A/duck/Vietnam/317/2005(H5N1)) hemagglutinin (HA) gene, partial cds.
gi|93008328|gb|DQ497690| /Avian/4(HA)/H5N1/Viet Nam/2003/// Influenza A virus (A/mallard/Vietnam/3/2003(H5N1)) hemagglutinin (HA) gene, partial cds.
gi|93008330|gb|DQ497691| /Avian/4(HA)/H5N1/Viet Nam/2003/// Influenza A virus (A/chicken/Vietnam/4/2003(H5N1)) hemagglutinin (HA) gene, partial cds.
gi|109727317|gb|DQ666146| /Human/4(HA)/H5N1/Djibouti/2006/// Influenza A virus (A/Djibouti/5691NAMRU3/2006(H5N1)) hemagglutinin (HA) gene, partial cds.
gi|93008254|gb|DQ497653| /Avian/4(HA)/H5N1/Indonesia/2004/// Influenza A virus (A/chicken/Purwakarta/BBVet-IV/2004(H5N1)) hemagglutinin (HA) gene, partial cds.
gi|93008256|gb|DQ497654| /Avian/4(HA)/H5N1/Indonesia/2004/// Influenza A virus (A/quail/Tasikmalaya/BPPV4/2004(H5N1)) hemagglutinin (HA) gene, partial cds.
gi|93008258|gb|DQ497655| /Avian/4(HA)/H5N1/Indonesia/2004/// Influenza A virus (A/chicken/Bangli Bali/BBPV6-1/2004(H5N1)) hemagglutinin (HA) gene, partial cds.
gi|93008260|gb|DQ497656| /Avian/4(HA)/H5N1/Indonesia/2004/// Influenza A virus (A/chicken/Bangli Bali/BBPV6-2/2004(H5N1)) hemagglutinin (HA) gene, partial cds.
gi|93008262|gb|DQ497657| /Avian/4(HA)/H5N1/Indonesia/2004/// Influenza A virus (A/chicken/Jembrana/BPPV6/2004(H5N1)) hemagglutinin (HA) gene, partial cds.
gi|93008344|gb|DQ497698| /Avian/4(HA)/H5N1/Viet Nam/2004/// Influenza A virus (A/chicken/Vietnam/52/2004(H5N1)) hemagglutinin (HA) gene, partial cds.
gi|93008266|gb|DQ497659| /Avian/4(HA)/H5N1/Indonesia/2005/// Influenza A virus (A/duck/Parepare/BBVM/2005(H5N1)) hemagglutinin (HA) gene, partial cds.
gi|93008268|gb|DQ497660| /Avian/4(HA)/H5N1/Indonesia/2004/// Influenza A virus (A/chicken/Kupang-2-NTT/BPPV6/2004(H5N1)) hemagglutinin (HA) gene, partial cds.
gi|93008270|gb|DQ497661| /Avian/4(HA)/H5N1/Indonesia/2004/// Influenza A virus (A/chicken/Kupang-3-NTT/BPPV6/2004(H5N1)) hemagglutinin (HA) gene, partial cds.
gi|93008272|gb|DQ497662| /Avian/4(HA)/H5N1/Indonesia/2004/// Influenza A virus (A/chicken/Kupang-1-NTT/BPPV6/2004(H5N1)) hemagglutinin (HA) gene, partial cds.
gi|93008274|gb|DQ497663| /Avian/4(HA)/H5N1/Indonesia/2004/// Influenza A virus (A/chicken/Pangkalpinang/BPPV3/2004(H5N1)) hemagglutinin (HA) gene, partial cds.
gi|93008356|gb|DQ497704| /Avian/4(HA)/H5N1/Viet Nam/2005/// Influenza A virus (A/duck/Vietnam/376/2005(H5N1)) hemagglutinin (HA) gene, partial cds.
gi|93008278|gb|DQ497665| /Avian/4(HA)/H5N1/Indonesia/2005/// Influenza A virus (A/chicken/Simalanggang/BPPVI/2005(H5N1)) hemagglutinin (HA) gene, partial cds.
gi|93008280|gb|DQ497666| /Avian/4(HA)/H5N1/Indonesia/2005/// Influenza A virus (A/chicken/Tebing Tinggi/BPPVI/2005(H5N1)) hemagglutinin (HA) gene, partial cds.
gi|93008282|gb|DQ497667| /Avian/4(HA)/H5N1/Indonesia/2005/// Influenza A virus (A/chicken/Dairi/BPPVI/2005(H5N1)) hemagglutinin (HA) gene, partial cds.
gi|93008284|gb|DQ497668| /Avian/4(HA)/H5N1/Indonesia/2005/// Influenza A virus (A/chicken/Deli Serdang/BPPVI/2005(H5N1)) hemagglutinin (HA) gene, partial cds.
gi|93008286|gb|DQ497669| /Avian/4(HA)/H5N1/Indonesia/2005/// Influenza A virus (A/chicken/Tarutung/BPPVI/2005(H5N1)) hemagglutinin (HA) gene, partial cds.
gi|93008288|gb|DQ497670| /Avian/4(HA)/H5N1/Viet Nam/2003/// Influenza A virus (A/duck/Vietnam/15/2003(H5N1)) hemagglutinin (HA) gene, partial cds.
gi|93008290|gb|DQ497671| /Avian/4(HA)/H5N1/Viet Nam/2003/// Influenza A virus (A/mallard/Vietnam/16/2003(H5N1)) hemagglutinin (HA) gene, partial cds.
gi|93008292|gb|DQ497672| /Avian/4(HA)/H5N1/Viet Nam/2003/// Influenza A virus (A/duck/Vietnam/17/2003(H5N1)) hemagglutinin (HA) gene, partial cds.
gi|93008294|gb|DQ497673| /Avian/4(HA)/H5N1/Viet Nam/2004/// Influenza A virus (A/duck/Vietnam/40/2004(H5N1)) hemagglutinin (HA) gene, partial cds.
gi|93008296|gb|DQ497674| /Avian/4(HA)/H5N1/Viet Nam/2005/// Influenza A virus (A/duck/Vietnam/272/2005(H5N1)) hemagglutinin (HA) gene, partial cds.
gi|93008378|gb|DQ497715| /Avian/4(HA)/H5N1/Viet Nam/2004/// Influenza A virus (A/quail/Vietnam/177/2004(H5N1)) hemagglutinin (HA) gene, partial cds.
gi|84797255|gb|DQ320936| /Avian/4(HA)/H5N1/Viet Nam/2005/// Influenza A virus (A/duck/Vietnam/S654/2005(H5N1)) hemagglutinin (HA) gene, partial cds.

gi|84797257|gb|DQ320937| /Avian/4(HA)/H5N1/Viet Nam/2004/// Influenza A virus (A/chicken/Vietnam/DT171/2004(H5N1)) hemagglutinin (HA) gene, partial cds.
gi|93008384|gb|DQ497718| /Avian/4(HA)/H5N1/Viet Nam/2004/// Influenza A virus (A/chicken/Vietnam/132/2004(H5N1)) hemagglutinin (HA) gene, partial cds.
gi|93008386|gb|DQ497719| /Human/4(HA)/H5N1/Viet Nam/2004/// Influenza A virus (A/Vietnam/CL01/2004(H5N1)) hemagglutinin (HA) gene, partial cds.
gi|93008308|gb|DQ497680| /Avian/4(HA)/H5N1/Viet Nam/2003/// Influenza A virus (A/mallard/Vietnam/21/2003(H5N1)) hemagglutinin (HA) gene, partial cds.
gi|93008310|gb|DQ497681| /Avian/4(HA)/H5N1/Viet Nam/2003/// Influenza A virus (A/chicken/Vietnam/28/2003(H5N1)) hemagglutinin (HA) gene, partial cds.
gi|93008312|gb|DQ497682| /Avian/4(HA)/H5N1/Viet Nam/2003/// Influenza A virus (A/chicken/Vietnam/30/2003(H5N1)) hemagglutinin (HA) gene, partial cds.
gi|93008314|gb|DQ497683| /Avian/4(HA)/H5N1/Viet Nam/2004/// Influenza A virus (A/duck/Vietnam/48/2004(H5N1)) hemagglutinin (HA) gene, partial cds.
gi|85062798|gb|DQ334776| /Avian/4(HA)/H5N1/Thailand/2005/// Influenza A virus (A/chicken/Thailand/Nontaburi/CK-162/2005(H5N1)) hemagglutinin gene, complete cds.
gi|93008398|gb|DQ497725| /Human/4(HA)/H5N1/Viet Nam/2004/// Influenza A virus (A/Vietnam/CL100/2004(H5N1)) hemagglutinin (HA) gene, partial cds.
gi|93008320|gb|DQ497686| /Avian/4(HA)/H5N1/Viet Nam/2004/// Influenza A virus (A/duck/Vietnam/N-XX/2004(H5N1)) hemagglutinin (HA) gene, partial cds.
gi|93008322|gb|DQ497687| /Avian/4(HA)/H5N1/Viet Nam/2004/// Influenza A virus (A/chicken/Vietnam/32/2004(H5N1)) hemagglutinin (HA) gene, partial cds.
gi|85062568|gb|DQ343152| /Avian/4(HA)/H5N1/China/2002/// Influenza A virus (A/chicken/Hebei/108/02(H5N1)) hemagglutinin (HA) gene, complete cds.
gi|85376991|gb|DQ343502| /Avian/4(HA)/H5N1/Russia/2005/11/26/ Influenza A virus (A/Cygnus olor/Astrakhan/Ast05-2-2/2005(H5N1)) hemagglutinin (HA) gene, complete cds.
gi|95116642|gb|DQ515984| /Avian/4(HA)/H5N1/Czech Republic/2006/03// Influenza A virus (A/Cygnus olor/Czech Republic/5170/2006 (H5N1)) hemagglutinin gene, complete cds.
gi|86211366|gb|DQ358746| /Avian/4(HA)/H5N1/Russia/2005/11/26/ Influenza A virus (A/Cygnus olor/Astrakhan/Ast05-2-3/2005(H5N1)) hemagglutinin (HA) gene, complete cds.
gi|110273419|gb|DQ838516| /Avian/4(HA)/H5N1/Niger/2006/05// Influenza A virus (A/chicken/Niger/2130-8/2006(H5N1)) hemagglutinin (HA) gene, complete cds.
gi|108782501|gb|DQ644955| /Avian/4(HA)/H5N1/Indonesia/2004/// Influenza A virus (A/chicken/Denpasar/01/2004(H5N1)) hemagglutinin (HA) gene, partial cds.
gi|108782503|gb|DQ644956| /Avian/4(HA)/H5N1/Indonesia/2005/// Influenza A virus (A/chicken/Denpasar/02/2005(H5N1)) hemagglutinin (HA) gene, partial cds.
gi|93008338|gb|DQ497695| /Avian/4(HA)/H5N1/Viet Nam/2004/// Influenza A virus (A/chicken/Vietnam/135/2004(H5N1)) hemagglutinin (HA) gene, partial cds.
gi|93008340|gb|DQ497696| /Avian/4(HA)/H5N1/Viet Nam/2004/// Influenza A virus (A/chicken/Vietnam/149/2004(H5N1)) hemagglutinin (HA) gene, partial cds.
gi|93008342|gb|DQ497697| /Avian/4(HA)/H5N1/Viet Nam/2005/// Influenza A virus (A/duck/Vietnam/286/2005(H5N1)) hemagglutinin (HA) gene, partial cds.
gi|109692765|gb|DQ650659| /Avian/4(HA)/H5N1/Ukraine/2005/// Influenza A virus (A/chicken/Crimea/04/2005(H5N1)) hemagglutinin (HA) mRNA, complete cds.
gi|109692773|gb|DQ650663| /Avian/4(HA)/H5N1/Ukraine/2005/// Influenza A virus (A/chicken/Crimea/08/2005(H5N1)) hemagglutinin (HA) mRNA, complete cds.
gi|105914725|gb|DQ659113| /Avian/4(HA)/H5N1/Niger/2006/02// Influenza A virus (A/duck/Niger/914/2006(H5N1)) hemagglutinin (HA), partial cds.
gi|87137936|gb|DQ371928| /Human/4(HA)/H5N1/China/2005/// Influenza A virus (A/Anhui/1/2005(H5N1)) hemagglutinin (HA) gene, complete cds.
gi|93008352|gb|DQ497702| /Avian/4(HA)/H5N1/Viet Nam/2004/// Influenza A virus (A/duck/Vietnam/148/2004(H5N1)) hemagglutinin (HA) gene, partial cds.
gi|93008354|gb|DQ497703| /Avian/4(HA)/H5N1/Viet Nam/2005/// Influenza A virus (A/chicken/Vietnam/398/2005(H5N1)) hemagglutinin (HA) gene, partial cds.
gi|107785189|gb|DQ661910| /Avian/4(HA)/H5N1/Afghanistan/2006/03// Influenza A virus (A/chicken/Afghanistan/1207/2006(H5N1)) hemagglutinin (HA) gene, partial cds.
gi|88714395|gb|DQ389158| /Avian/4(HA)/H5N1/Russia/2005/// Influenza A virus (A/Cygnus olor/Astrakhan/Ast05-2-1/2005(H5N1)) hemagglutinin (HA) gene, complete cds.
gi|93008360|gb|DQ497706| /Avian/4(HA)/H5N1/Viet Nam/2004/// Influenza A virus (A/chicken/Vietnam/260/2004(H5N1)) hemagglutinin (HA) gene, partial cds.
gi|93008362|gb|DQ497707| /Avian/4(HA)/H5N1/Viet Nam/2004/// Influenza A virus (A/goose/Vietnam/264/2004(H5N1)) hemagglutinin (HA) gene, partial cds.
gi|93008364|gb|DQ497708| /Avian/4(HA)/H5N1/Viet Nam/2005/// Influenza A virus (A/duck/Vietnam/283/2005(H5N1)) hemagglutinin (HA) gene, partial cds.
gi|93008366|gb|DQ497709| /Avian/4(HA)/H5N1/Viet Nam/2005/// Influenza A virus (A/duck/Vietnam/557/2005(H5N1)) hemagglutinin (HA) gene, partial cds.
gi|93008368|gb|DQ497710| /Avian/4(HA)/H5N1/Viet Nam/2004/// Influenza A virus (A/chicken/Vietnam/53/2004(H5N1)) hemagglutinin (HA) gene, partial cds.
gi|93008370|gb|DQ497711| /Avian/4(HA)/H5N1/Viet Nam/2005/// Influenza A virus (A/quail/Vietnam/282/2005(H5N1)) hemagglutinin (HA) gene, partial cds.
gi|84797247|gb|DQ320932| /Avian/4(HA)/H5N1/Indonesia/2005/// Influenza A virus (A/chicken/Bantul/BBVet-I/2005(H5N1)) hemagglutinin (HA) gene, partial cds.
gi|84797249|gb|DQ320933| /Avian/4(HA)/H5N1/Indonesia/2005/// Influenza A virus (A/chicken/Wajo/BBVM/2005(H5N1)) hemagglutinin (HA) gene, partial cds.
gi|84797251|gb|DQ320934| /Avian/4(HA)/H5N1/Malaysia/2004/// Influenza A virus (A/chicken/Malaysia/5858/2004(H5N1)) hemagglutinin (HA) gene, partial cds.
gi|47834887|gb|AY575879| /Avian/4(HA)/H5N1/Hong Kong/2002/// Influenza A virus (A/Ck/HK/409.1/02 (H5N1)) hemagglutinin (HA) gene, partial cds.
gi|47834885|gb|AY575878| /Avian/4(HA)/H5N1/Hong Kong/2002/// Influenza A virus (A/Ck/HK/96.1/02 (H5N1)) hemagglutinin (HA) gene, partial cds.
gi|85062564|gb|DQ343150| /Avian/4(HA)/H5N1/China/2005/// Influenza A virus (A/chicken/Hebei/326/2005(H5N1)) hemagglutinin (HA) gene, complete cds.
gi|98418223|gb|DQ530173| /Canine/4(HA)/H5N1/Thailand/2004/// Influenza A Virus (A/dog/Thailand-Suphanburi/KU-08/04(H5N1)) hemagglutinin gene, partial cds.
gi|110613449|gb|DQ840519| /Avian/4(HA)/H5N1/Russia/2005/// Influenza A virus (A/chicken/Tula/Russia/Oct-5/2005(H5N1)) hemagglutinin (HA) gene, partial cds.
gi|93008388|gb|DQ497720| /Human/4(HA)/H5N1/Viet Nam/2004/// Influenza A virus (A/Vietnam/CL02/2004(H5N1)) hemagglutinin (HA) gene, partial cds.
gi|93008390|gb|DQ497721| /Human/4(HA)/H5N1/Viet Nam/2004/// Influenza A virus (A/Vietnam/CL17/2004(H5N1)) hemagglutinin (HA) gene, partial cds.
gi|110631382|gb|DQ861999| /Avian/4(HA)/H5N1/Sudan/2006/05// Influenza A virus (A/chicken/Sudan/2115-12/2006(H5N1)) hemagglutinin (HA) gene, complete cds.
gi|85062782|gb|DQ334768| /Avian/4(HA)/H5N1/Thailand/2005/// Influenza A virus (A/quail/Thailand/Nakhon Pathom/QA-161/2005(H5N1)) hemagglutinin gene, complete cds.
gi|4240447|gb|AF082040| /Avian/4(HA)/H5N1/USA/1981/// Influenza A virus (A/duck/Minnesota/1525/81(H5N1)) hemagglutinin H5 mRNA, partial cds.
gi|93008396|gb|DQ497724| /Human/4(HA)/H5N1/Viet Nam/2004/// Influenza A virus (A/Vietnam/CL36/2004(H5N1)) hemagglutinin (HA) gene, partial cds.
gi|93008238|gb|DQ497645| /Avian/4(HA)/H5N1/Indonesia/2003/// Influenza A virus (A/chicken/Pekalongan/BPPV4/2003(H5N1)) hemagglutinin (HA) gene, partial cds.
gi|85062566|gb|DQ343151| /Avian/4(HA)/H5N1/China/2001/// Influenza A virus (A/chicken/Hebei/718/2001(H5N1)) hemagglutinin (HA) gene, complete cds.
gi|93008402|gb|DQ497727| /Human/4(HA)/H5N1/Viet Nam/2005/// Influenza A virus (A/Vietnam/CL115/2005(H5N1)) hemagglutinin (HA) gene, partial cds.
gi|93008404|gb|DQ497728| /Human/4(HA)/H5N1/Viet Nam/2005/// Influenza A virus (A/Vietnam/CL119/2005(H5N1)) hemagglutinin (HA) gene, partial cds.
gi|93008406|gb|DQ497729| /Human/4(HA)/H5N1/Viet Nam/2005/// Influenza A virus (A/Vietnam/CL2009/2005(H5N1)) hemagglutinin (HA) gene, partial cds.
gi|60482|gb|X07869| /Avian/4(HA)/H5N1/United Kingdom/1959/// Influenza A virus (A/chicken/Scotland/59(H5N1)) mRNA for haemagglutinin precursor
gi|110273421|gb|DQ838517| /Avian/4(HA)/H5N1/Niger/2006/05// Influenza A virus (A/chicken/Niger/2130-7/2006(H5N1)) hemagglutinin (HA) gene, complete cds.
gi|87204345|gb|DQ363923| /Avian/4(HA)/H5N1/Russia/2005/// Influenza A virus (A/Cygnus olor/Astrakhan/Ast05-2-7/2005(H5N1)) hemagglutinin (HA) gene, complete cds.
gi|86278718|gb|DQ364996| /Avian/4(HA)/H5N1/Russia/2005/11/26/ Influenza A virus (A/Cygnus olor/Astrakhan/Ast05-2-6/2005(H5N1)) hemagglutinin (HA) gene, complete cds.
gi|86278736|gb|DQ365004| /Avian/4(HA)/H5N1/Russia/2005/11/26/ Influenza A virus (A/Cygnus olor/Astrakhan/Ast05-2-5/2005(H5N1)) hemagglutinin (HA) gene, complete cds.
gi|108782507|gb|DQ644958| /Avian/4(HA)/H5N1/Indonesia/2005/// Influenza A virus (A/duck/Buleleng-Bali/04/2005(H5N1)) hemagglutinin (HA) gene, partial cds.
gi|86753757|gb|DQ366314| /Avian/4(HA)/H5N1/Viet Nam/2005/// Influenza A virus (A/goose/Vietnam/3/05(H5N1)) hemagglutinin mRNA, complete cds.
gi|110631386|gb|DQ862001| /Avian/4(HA)/H5N1/Egypt/2006/06// Influenza A virus (A/chicken/Egypt/2253-1/2006(H5N1)) hemagglutinin (HA) gene, complete cds.
gi|93008264|gb|DQ497658| /Avian/4(HA)/H5N1/Indonesia/2004/// Influenza A virus (A/chicken/Mangarai-NTT/BPPV6/2004(H5N1)) hemagglutinin (HA) gene, partial cds.
gi|110333722|gb|DQ837587| /Avian/4(HA)/H5N1/Egypt/2006/// Influenza A virus (A/Chicken/Egypt/5610NAMRU3-F3/2006(H5N1)) hemagglutinin (HA) gene, partial cds.
gi|110333724|gb|DQ837588| /Avian/4(HA)/H5N1/Egypt/2006/// Influenza A virus (A/Chicken/Egypt/5611NAMRU3-AN/2006(H5N1)) hemagglutinin (HA) gene, partial cds.
gi|87137938|gb|DQ371929| /Human/4(HA)/H5N1/China/2005/// Influenza A virus (A/Anhui/2/2005(H5N1)) hemagglutinin (HA) gene, complete cds.
gi|87137940|gb|DQ371930| /Human/4(HA)/H5N1/China/2005/// Influenza A virus (A/Guangxi/1/2005(H5N1)) hemagglutinin (HA) gene, complete cds.
gi|87042573|gb|DQ372591| /Human/4(HA)/H5N1/Thailand/2005/// Influenza A virus (A/Thailand/NK165/2005(H5N1)) hemagglutinin (HA) gene, partial cds.
gi|110084604|gb|DQ659679| /Avian/4(HA)/H5N1/Germany/2006/// Influenza A virus (A/common bussard/Bavaria/2/2006(H5N1)) hemagglutinin (HA) gene, complete cds.
gi|109630638|gb|DQ767725| /Avian/4(HA)/H5N1/China/2004/// Influenza A virus (A/chicken/Shandong/K01/2004(H5N1)) segment 4, complete sequence.
gi|89077659|gb|DQ399547| /Avian/4(HA)/H5N1/Russia/2005/11// Influenza A virus (A/Cygnus olor/Astrakhan/Ast05-2-9/2005(H5N1)) hemagglutinin (HA) gene, complete cds.
gi|110273403|gb|DQ838508| /Avian/4(HA)/H5N1/Italy/2005/// Influenza A virus (A/mallard/Italy/3401/2005(H5N1)) hemagglutinin (HA) gene, partial cds.
gi|110333726|gb|DQ837589| /Avian/4(HA)/H5N1/Egypt/2006/// Influenza A virus (A/Chicken/Egypt/5612NAMRU3-S/2006(H5N1)) hemagglutinin (HA) gene, partial cds.
gi|94961242|gb|DQ412997| /Avian/4(HA)/H5N1/Italy/2006/02/10/ Influenza A virus (A/Cygnus olor/Italy/742/2006(H5N1)) hemagglutinin (HA) gene, partial cds.
gi|91984134|gb|DQ095627| /Avian/4(HA)/H5N1/China/2005/// Influenza A virus (A/Quail/Shantou/911/05(H5N1)) hemagglutinin (HA) gene, partial cds.
gi|101918580|gb|AB259712| /Avian/4(HA)/H5N1/Japan/2004/// Influenza A virus (A/duck/Hokkaido/Vac-1/04(H5N1)) genomic RNA, segment 4, complete sequence.
gi|47156278|gb|AY585362| /Avian/4(HA)/H5N1/China/2002/// Influenza A virus (A/duck/Guangdong/22/2002(H5N1)) hemagglutinin (HA) mRNA, complete cds.
gi|109452300|gb|AB263192| /Avian/4(HA)/H5N1/Mongolia/2001/// Influenza A virus (A/R(duck/Mongolia/54/01-duck/Mongolia/47/01) (H5N1)) genomic RNA, segment 4, complete sequence.
gi|93008376|gb|DQ497714| /Avian/4(HA)/H5N1/Viet Nam/2004/// Influenza A virus (A/chicken/Vietnam/134/2004(H5N1)) hemagglutinin (HA) gene, partial cds.
gi|108671042|gb|DQ659326| /Avian/4(HA)/H5N1/Mongolia/2005/// Influenza A virus (St Jude H5N1 influenza seed virus 163243) hemagglutinin (HA) gene, complete cds.
gi|93008380|gb|DQ497716| /Avian/4(HA)/H5N1/Viet Nam/2004/// Influenza A virus (A/duck/Vietnam/258/2004(H5N1)) hemagglutinin (HA) gene, partial cds.
gi|86753763|gb|DQ366338| /Avian/4(HA)/H5N1/China/2004/// Influenza A virus (A/duck/Guangxi/13/2004(H5N1)) hemagglutinin mRNA, complete cds.
gi|110333728|gb|DQ837590| /Avian/4(HA)/H5N1/Egypt/2006/// Influenza A virus (A/Turkey/Egypt/5613NAMRU3-T/2006(H5N1)) hemagglutinin (HA) gene, partial cds.
gi|60702|gb|X07826| /Avian/4(HA)/H5N1/United Kingdom/1959/// Influenza A virus (A/chicken/Scotland/59(H5N1)) HA gene for hemagglutinin, genomic RNA
gi|108782511|gb|DQ676830| /Avian/4(HA)/H5N1/Russia/2006/// Influenza A virus (A/chicken/Mahachkala/05/2006(H5N1)) hemagglutinin (HA) mRNA, complete cds.
gi|110287938|gb|DQ836043| /Avian/4(HA)/H5N1/China/2004/// Influenza A virus (A/goose/Huadong/220/2004(H5N1)) hemagglutinin (HA) gene, complete cds.
gi|4240453|gb|AF082043| /Avian/4(HA)/H5N1/USA/1983/// Influenza A virus (A/gull/Pennsylvania/4175/83(H5N1)) hemagglutinin H5 mRNA, partial cds.
gi|93008394|gb|DQ497723| /Human/4(HA)/H5N1/Viet Nam/2004/// Influenza A virus (A/Vietnam/CL26/2004(H5N1)) hemagglutinin (HA) gene, partial cds.
gi|108782509|gb|DQ644959| /Avian/4(HA)/H5N1/Indonesia/2005/// Influenza A virus (A/duck/Badung-Bali/05/2005(H5N1)) hemagglutinin (HA) gene, partial cds.
gi|108671044|gb|DQ659327| /Avian/4(HA)/H5N1/China/2005/// Influenza A virus (St Jude H5N1 influenza seed virus 163222) hemagglutinin (HA) gene, complete cds.
gi|110631390|gb|DQ862003| /Avian/4(HA)/H5N1/Sudan/2006/05// Influenza A virus (A/chicken/Sudan/1784/2006(H5N1)) hemagglutinin (HA) gene, partial cds.
gi|93008374|gb|DQ497713| /Avian/4(HA)/H5N1/Viet Nam/2004/// Influenza A virus (A/chicken/Vietnam/159/2004(H5N1)) hemagglutinin (HA) gene, partial cds.
gi|47834879|gb|AY575875| /Avian/4(HA)/H5N1/Hong Kong/2002/// Influenza A virus (A/Ck/HK/31.4/02 (H5N1)) hemagglutinin (HA) gene, partial cds.

gi|67107296|gb|S68489| /Avian/4(HA)/H5N1/United Kingdom/1991/// hemagglutinin [H5N1 avian influenza virus, A/turkey/England/50-92/91, Genomic, 1773 nt]
gi|108782519|gb|DQ676834| /Avian/4(HA)/H5N1/Russia/2006/// Influenza A virus (A/chicken/Krasnodar/01/2006(H5N1)) hemagglutinin (HA) mRNA, complete cds.
gi|93008382|gb|DQ497717| /Avian/4(HA)/H5N1/Viet Nam/2005/// Influenza A virus (A/duck/Vietnam/543/2005(H5N1)) hemagglutinin (HA) gene, partial cds.
gi|108782531|gb|DQ676840| /Avian/4(HA)/H5N1/Russia/2005/// Influenza A virus (A/goose/Krasnoozerka/627/2005(H5N1)) hemagglutinin (HA) mRNA, complete cds.
gi|108782505|gb|DQ644957| /Avian/4(HA)/H5N1/Indonesia/2005/// Influenza A virus (A/duck/Jembrana-Bali/03/2005(H5N1) hemagglutinin (HA) gene, partial cds.
gi|93008372|gb|DQ497712| /Avian/4(HA)/H5N1/Viet Nam/2005/// Influenza A virus (A/chicken/Vietnam/393/2005(H5N1)) hemagglutinin (HA) gene, partial cds.
gi|110631384|gb|DQ862000| /Avian/4(HA)/H5N1/Sudan/2006/05// Influenza A virus (A/chicken/Sudan/2115-9/2006(H5N1)) hemagglutinin (HA) gene, complete cds.
gi|58618437|gb|AY818135| /Human/4(HA)/H5N1/Viet Nam/2004/// Influenza A virus (A/Viet Nam/1203/2004(H5N1)) hemagglutinin HA gene, complete cds.
gi|50296052|gb|AY651334| /Human/4(HA)/H5N1/Viet Nam/2004/// Influenza A virus (A/Viet Nam/1203/2004(H5N1)) hemagglutinin (HA) gene, partial cds.
gi|2865379|gb|AF028709| /Human/4(HA)/H5N1/Hong Kong/1997/// Influenza A virus (A/HongKong/156/97(H5N1)) hemagglutinin mRNA, complete cds.
gi|2833656|gb|AF036356| /Human/4(HA)/H5N1/Hong Kong/1997/// Influenza A virus (A/HongKong/156/97(H5N1)) H5 hemagglutinin (HA) gene, partial cds.
gi|3335402|gb|AF046080| /Avian/4(HA)/H5N1/Hong Kong/1997/// Influenza A virus (A/Chicken/Hong Kong/220/97 (H5N1)) hemagglutinin subtype H5 (H5) gene, complete cds.
gi|3335420|gb|AF046088| /Human/4(HA)/H5N1/Hong Kong/1997/// Influenza A virus (A/Hong Kong/156/97(H5N1)) hemagglutinin subtype H5 (H5) gene, complete cds.
gi|3421251|gb|AF046096| /Human/4(HA)/H5N1/Hong Kong/1997/// Influenza A virus (A/Hong Kong/481/97(H5N1)) hemagglutinin subtype H5 (H5) gene, complete cds.
gi|3421255|gb|AF046097| /Human/4(HA)/H5N1/Hong Kong/1997/// Influenza A Virus (A/Hong Kong/483/97(H5N1)) hemagglutinin subtype H5 (H5) gene, complete cds.
gi|3421259|gb|AF046098| /Human/4(HA)/H5N1/Hong Kong/1997/// Influenza A virus (A/Hong Kong/482/97(H5N1)) hemagglutinin subtype H5 (H5) gene, complete cds.
gi|3421263|gb|AF046099| /Avian/4(HA)/H5N1/Hong Kong/1997/// Influenza A virus (A/Chicken/Hong Kong/728/97 (H5N1)) hemagglutinin subtype H5 (H5) gene, complete cds.
gi|3068720|gb|AF057291| /Avian/4(HA)/H5N1/Hong Kong/1997/// Influenza A virus (A/chicken/Hong Kong/258/97(H5N1)) hemagglutinin mRNA, complete cds.
gi|4240435|gb|AF082034| /Avian/4(HA)/H5N1/Hong Kong/1997/// Influenza A virus (A/Chicken/Hong Kong/728/97 (H5N1)) hemagglutinin H5 mRNA, complete cds.
gi|4240437|gb|AF082035| /Avian/4(HA)/H5N1/Hong Kong/1997/// Influenza A virus (A/Chicken/Hong Kong/786/97 (H5N1)) hemagglutinin H5 mRNA, complete cds.
gi|8307800|gb|AF084279| /Human/4(HA)/H5N1/Hong Kong/1997/// Influenza A virus (A/HongKong/481/97(H5N1)) segment 4 hemagglutinin (ha) gene, partial cds.
gi|8307802|gb|AF084280| /Human/4(HA)/H5N1/Hong Kong/1997/// Influenza A virus (A/HongKong/483/97(H5N1)) segment 4 hemagglutinin (ha) gene, partial cds.
gi|8307804|gb|AF084281| /Human/4(HA)/H5N1/Hong Kong/1997/// Influenza A virus (A/HongKong/486/97(H5N1)) segment 4 hemagglutinin (ha) gene, partial cds.
gi|5813856|gb|AF084532| /Human/4(HA)/H5N1/Hong Kong/1997/// Influenza A virus (A/HongKong/485/97(H5N1)) segment 4 hemagglutinin (HA) gene, partial cds.
gi|6048752|gb|AF098541| /Avian/4(HA)/H5N1/Hong Kong/1997/// Influenza A virus (A/Chicken/Hong Kong/y385/97 (H5N1)) hemagglutinin gene, partial cds.
gi|6048754|gb|AF098542| /Avian/4(HA)/H5N1/Hong Kong/1997/// Influenza A virus (A/Chicken/Hong Kong/y388/97 (H5N1)) hemagglutinin gene, partial cds.
gi|6048756|gb|AF098543| /Avian/4(HA)/H5N1/Hong Kong/1997/// Influenza A virus (A/Duck/Hong Kong/p46/97 (H5N1)) hemagglutinin gene, partial cds.
gi|6048760|gb|AF098545| /Avian/4(HA)/H5N1/Hong Kong/1997/// Influenza A virus (A/Goose/Hong Kong/w355/97 (H5N1)) hemagglutinin gene, partial cds.
gi|6048762|gb|AF098546| /Avian/4(HA)/H5N1/Hong Kong/1997/// Influenza A virus (A/Silky Chicken/Hong Kong/p17/97 (H5N1)) hemagglutinin gene, partial cds.
gi|4457115|gb|AF102671| /Human/4(HA)/H5N1/Hong Kong/1997/// Influenza A virus (A/Hong Kong/486/97(H5N1)) hemagglutinin (HA) gene, partial cds.
gi|4457116|gb|AF102672| /Human/4(HA)/H5N1/Hong Kong/1997/// Influenza A virus (A/Hong Kong/488/97(H5N1)) hemagglutinin (HA) gene, partial cds.
gi|4457117|gb|AF102673| /Human/4(HA)/H5N1/Hong Kong/1997/// Influenza A virus (A/Hong Kong/516/97(H5N1)) hemagglutinin (HA) gene, partial cds.
gi|4457118|gb|AF102674| /Human/4(HA)/H5N1/Hong Kong/1997/// Influenza A virus (A/Hong Kong/538/97(H5N1)) hemagglutinin (HA) gene, partial cds.
gi|4457119|gb|AF102675| /Human/4(HA)/H5N1/Hong Kong/1997/// Influenza A virus (A/Hong Kong/507/97(H5N1)) hemagglutinin (HA) gene, partial cds.
gi|4457120|gb|AF102676| /Human/4(HA)/H5N1/Hong Kong/1998/// Influenza A virus (A/HongKong/97/98(H5N1)) hemagglutinin (HA) gene, partial cds.
gi|4457121|gb|AF102677| /Human/4(HA)/H5N1/Hong Kong/1997/// Influenza A virus (A/Hong Kong/491/97(H5N1)) hemagglutinin (HA) gene, partial cds.
gi|4457122|gb|AF102678| /Human/4(HA)/H5N1/Hong Kong/1997/// Influenza A virus (A/Hong Kong/542/97(H5N1)) hemagglutinin (HA) gene, partial cds.
gi|4457123|gb|AF102679| /Human/4(HA)/H5N1/Hong Kong/1997/// Influenza A virus (A/Hong Kong/503/97(H5N1)) hemagglutinin (HA) gene, partial cds.
gi|4457124|gb|AF102680| /Human/4(HA)/H5N1/Hong Kong/1997/// Influenza A Virus (A/Hong Kong/532/97(H5N1)) hemagglutinin (HA) gene, partial cds.
gi|4457125|gb|AF102681| /Human/4(HA)/H5N1/Hong Kong/1997/// Influenza A virus (A/Hong Kong/485/97(H5N1)) hemagglutinin (HA) gene, partial cds.
gi|4457126|gb|AF102682| /Human/4(HA)/H5N1/Hong Kong/1997/// Influenza A virus (A/Hong Kong/514/97(H5N1)) hemagglutinin (HA) gene, partial cds.
gi|5805286|gb|AF144305| /Avian/4(HA)/H5N1/China/1996/// Influenza A virus (A/Goose/Guangdong/1/96(H5N1)) hemagglutinin (HA) gene, complete cds.
gi|5007022|gb|AF148678| /Avian/4(HA)/H5N1/China/1996/// Influenza A virus (A/goose/Guangdong/1/96/(H5N1)) hemagglutinin mRNA, complete cds.
